# Femtosecond to Microsecond Observation of Photochemical Pathways in Nitroaromatic Phototriggers Using Transient Absorption Spectroscopy

**DOI:** 10.1021/acs.jpca.4c02482

**Published:** 2024-07-11

**Authors:** William Whitaker, Deborin Ghosh, Partha Malakar, Gabriel Karras, Andrew J. Orr-Ewing

**Affiliations:** † School of Chemistry, University of Bristol, Cantock’s Close, Bristol BS8 1TS, U.K.; ‡ Central Laser Facility, Research Complex at Harwell, Science and Technology Facilities Council, Rutherford Appleton Laboratory, Harwell Oxford, Didcot, Oxfordshire OX11 0QX, U.K.

## Abstract

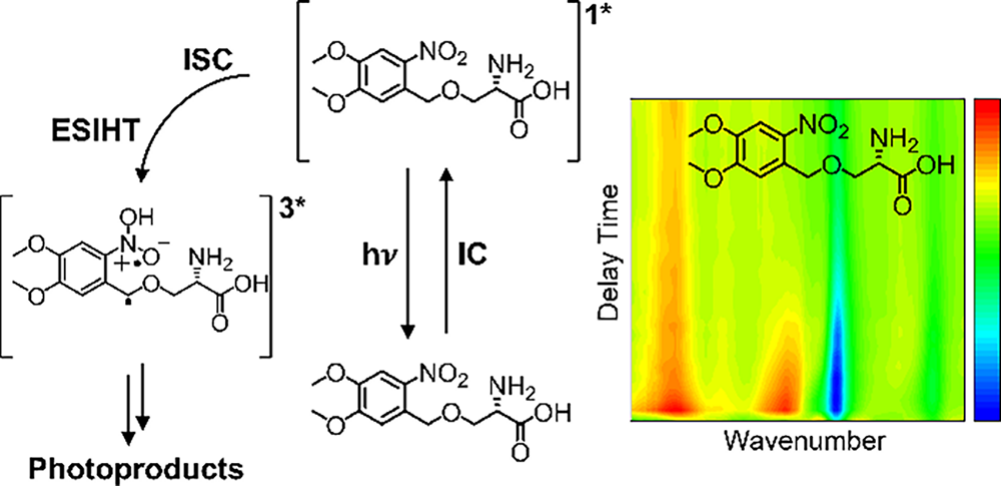

The synthetic accessibility and tolerance to structural modification of phototriggered compounds (PTs) based on the *ortho*- nitrobenzene (ONB) protecting group have encouraged a myriad of applications including optimization of biological activity, and supramolecular polymerization. Here, a combination of ultrafast transient absorption spectroscopy techniques is used to study the multistep photochemistry of two nitroaromatic phototriggers based on the ONB chromophore, *O*-(4,5-dimethoxy-2-nitrobenzyl)-l-serine (DMNB-Ser) and *O-*[(2-nitrophenyl)methyl]-l-tyrosine hydrochloride (NB-Tyr), in DMSO solutions on femtosecond to microsecond time scales following the absorption of UV light. From a common *nitro*-S_1_ excited state, the PTs can either undergo excited state intramolecular hydrogen transfer (ESIHT) to an *aci*-S_1_ isomer within the singlet state manifold, leading to direct S_1_ → S_0_ internal conversion through a conical intersection, or competitive intersystem crossing (ISC) to access the triplet state manifold on time scales of (1.93 ± 0.03) ps and (13.9 ± 1.2) ps for DMNB-Ser and NB-Tyr, respectively. Deprotonation of *aci*-T_1_ species to yield triplet anions is proposed to occur in both PTs, with an illustrative time constant of (9.4 ± 0.7) ns for DMNB-Ser. More than 75% of the photoexcited molecules return to their electronic ground states within 8 μs, either by direct S_1_ → S_0_ relaxation or anion reprotonation. Hence, upper limits to the quantum yields of photoproduct formation are estimated to be in the range of 13–25%. Mixed DMSO/H_2_O solvents show the influence of the environment on the observed photochemistry, for example, revealing two *nitro*-S_1_ lifetimes for DMNB-Ser in a 10:1 DMSO/H_2_O mixture of 1.95 ps and (10.1 ± 1.2) ps, which are attributed to different microsolvation environments.

## 1 Introduction

Phototriggered compounds (phototriggers, PTs), sometimes referred to as “caged” compounds, consist of a photolabile chromophore that is covalently bound to a chemically or biologically active group. While bound, the chromophore behaves as a protecting group, inhibiting the function of the active moiety often by restricting intermolecular interactions with external substrates.^1−5^ However, absorption of certain wavelengths of light initiates a specific bond cleavage that liberates the active molecule, facilitating a regain of function.^6−8^ Laser irradiation of PTs affords high spatial and temporal control over the reactivation process and can be used for site-specific drug delivery mechanisms,^9^ exploited in research to monitor specific biological processes,^6,10−12^ or to observe the kinetics of physiological processes, for example, in kinase photoreceptors.^13,14^ Furthermore, inactive (bound) forms of PTs can be activated directly at sites of interest, eliminating the influence of diffusion on observed kinetics and establishing a well-defined time zero in physiological response measurements.^15,16^

Common protecting groups include *ortho*- nitrobenzene (ONB) derivatives,^17−19^ benzoin,^20^ and *para-*hydroxyphenacyl (pHP);^21^ however any chromophore that releases an active species rapidly on light absorption, and ideally in high yield, is, in principle, a suitable PT candidate.^7,22^ Despite comparatively low quantum yields of photodeprotection (0.1–0.2),^8^ ONB PTs have been used extensively because of their synthetic accessibility, commercial availability, and tolerance to modification.^9,23−25^ ONB is reported as a protecting group for a variety of substrates including fluorouracil conjugated gold nanoparticles as anticancer drugs,^26^ amino acids including tyrosine^14,27,28^ and serine,^29^ and dopamine D2/D3 receptors.^30^ More recently, ONB PTs have been applied in the control of supramolecular polymerization of naphthalenediimide derivatives,^31^ modified with pH-sensitive moieties to develop protecting groups that are sensitive to their environment,^32^ and integrated into nucleic acid scaffolds for a variety of biological and materials applications.^33^ The 4,5-dimethoxy-2-nitrobenzyl (DMNB) protecting group is a derivative of ONB that requires lower photon energy to initiate photodeprotection compared to the parent compound.^34^ Activation of functional moieties using longer wavelengths of light is desirable, especially in biological applications where high-energy radiation can cause damage to samples or living tissue. Consequently, DMNB has become a popular choice of photoprotecting group in a variety of applications including studies to optimize the activity of biological processes,^35^ and light-induced micelle formation in block copolymers.^36^

**Figure 1:**
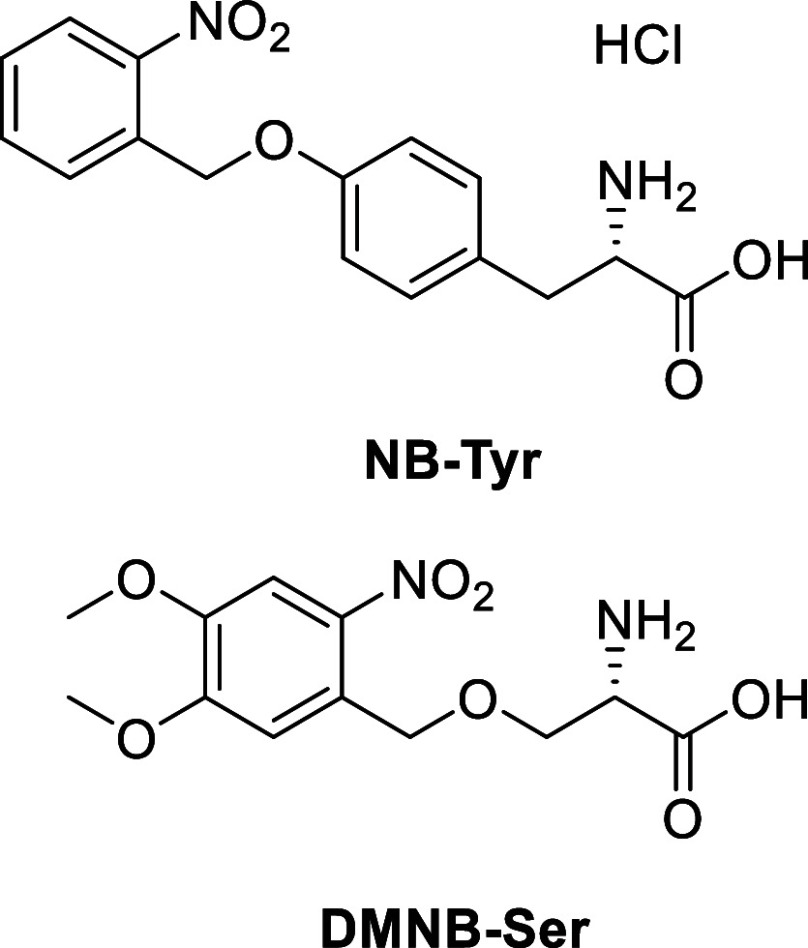
Chemical structures of the phototriggers studied in this work. Cleavage occurs across the C–O bond *ortho*- to the *nitro*- moiety.

Motivated by the myriad applications and efficacy of nitrobenzyl-type PTs, we have selected two biologically relevant compounds derived from ONB to investigate their mechanisms of photorelease. These two compounds are *O-*[(2-nitrophenyl)methyl]-l-tyrosine hydrochloride (NB-Tyr) and *O*-(4,5-dimethoxy-2nitrobenzyl)-l-serine (DMNB-Ser), with structures shown in Figure 1.

Individual components of the overall dynamics for ONB derivatives have been explored in prior studies, including subpicosecond internal conversion (IC) dynamics and excited state lifetimes of 4,5-dimethoxy-2-nitrobenzyl acetate.^25^ A prior time-resolved FTIR study of nitrophenyl ethers determined the rate constants for the decay of relevant intermediates in photodeprotection mechanisms,^37^ while an investigation of the photochemistry of *o*-nitrobenzyl compounds used a combination of time-resolved and steady-state spectroscopies.^38^ A comprehensive theoretical study of *o*-nitrobenzyl acetate used a high level of electronic structure theory to investigate the formation mechanisms and relative stability of its *aci-* isomers in the ground and electronically excited states.^8^

**Figure 2:**
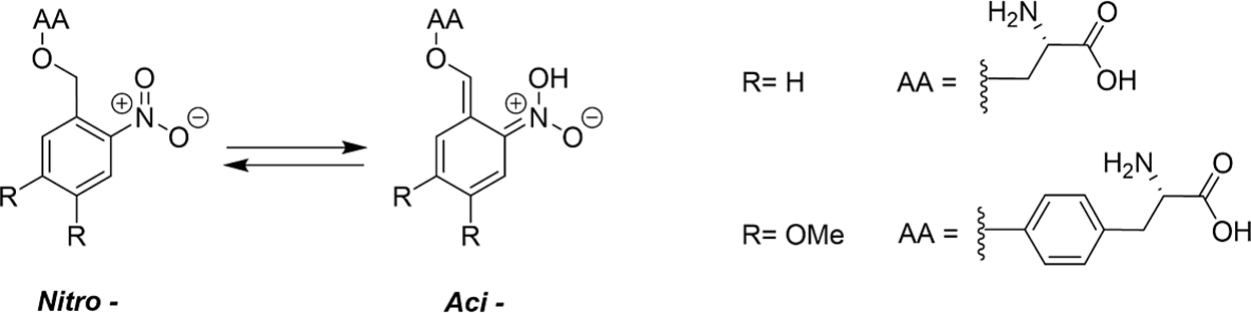
*Aci*/*nitro*- tautomerism for the nitroaromatic phototriggered compounds investigated in this study. AA denotes an amino-acid substituent. *Aci*- isomers can exist in four configurations depending on the substituent geometry about the C=C and C=N double bonds formed on ESIHT, taking the form of X,Y- *aci*- where X and Y refer to the E/Z stereochemistry of the C=C and C=N bonds, respectively. The E,Z-*aci* configuration is shown here.

Interpretation of the results from the photochemical studies of ONB PT compounds in solution presented here is guided by recent experimental and theoretical studies of related nitroaromatic chromophores including nitrobenzene^39−42^ and nitrophenols.^43,44^ Prior studies of these structurally simpler nitroaromatic compounds have revealed the importance of ultrafast intersystem crossing (ISC) and S_1_ to S_0_ IC pathways on the photochemical outcomes of these systems.^45−51^ Understanding this behavior of the core nitroaromatic chromophore proves important for unraveling the photochemical dynamics of the more complex ONB PT analogues. Of particular relevance in photodeprotection mechanisms is the observation of *aci*/*nitro* tautomerism exhibited by ONB compounds.^8,24,52−54^ The *nitro-* configuration is generally the more stable tautomeric form in the ground electronic state, but following UV photoabsorption, excited state intramolecular hydrogen transfer (ESIHT) can occur within either a singlet or triplet excited electronic state. Figure 2 illustrates this process: hydrogen transfer from the *ortho-* substituent to the *nitro-* moiety is accompanied by a redistribution of electron density about the chromophore such that new bond character is established.^43,55,56^ Calculations by Mewes and Dreuw indicate that, in most cases, the *nitro*- tautomers become unstable relative to their *aci-* isomers in the electronically excited states.^8^

Using transient electronic and transient vibrational absorption spectroscopy techniques, this study elucidates the IC, ISC, ESIHT, and subsequent dynamics of nitroaromatic phototriggers over subpicosecond to microsecond time scales immediately following near-UV photoexcitation. The influences of the solvent environment on the dynamics, and on the rates of photophysical and photochemical processes, are also evaluated to better understand and predict the behavior of PTs in a variety of conditions.

## 2 Methods

Solutions of DMNB-Ser (3–6.5 mM) and NB-Tyr (1.6 mM) were prepared in DMSO and DMSO-*d*_6_ for transient electronic absorption spectroscopy (TEAS) and transient vibrational absorption spectroscopy (TVAS), respectively. For measurements in mixed solvents, H_2_O/D_2_O was added stepwise to DMSO/DMSO-*d*_6_ solutions in intervals of 1 mL to yield solvent compositions that are 10:1 DMSO/DMSO-*d*_6_: H_2_O/D_2_O and 5:1 DMSO/DMSO-*d*_6_: H_2_O/D_2_O by volume. For time-resolved measurements of DMNB-Ser, an ultrafast 360 nm UV pump pulse was used to excite the sample electronically, whereas for NB-Tyr an ultrafast 285 nm UV pump pulse was selected. TEAS measurements made at the University of Bristol used a broadband white-light continuum (WLC) probe pulse generated by focusing 800 nm light on a rastered CaF_2_ window to obtain spectra at time delays from 100 fs to 3.5 ns with a resolution of 100 fs. Additional TEAS measurements acquired at the LIFEtime facility located at the Rutherford Appleton Laboratory used two separate WLC probe pulses spanning the regions 370–480 nm and 470–920 nm to observe dynamics over picosecond to microsecond time delays. TVAS measurements performed with LIFEtime used a pair of synchronized broadband IR pulses covering a 400 cm^–1^ spectral range when contiguously frequency-tuned. Only a single IR probe region is reported here for delay times extending into the microsecond regime.^57,58^ More complete descriptions of the experimental methods are available in section S1 of the Supporting Information.

To support the interpretation of our experimental results, density functional theory (DFT) and time-dependent density functional theory (TDDFT) calculations were performed at the ωB97XD/6-31+G(d) level of theory using the Tamm-Dancoff approximation (TDA). The range-separated hybrid functional ωB97XD was chosen for calculations as it should well-describe the inter- and intramolecular H atom transfer dynamics that have been adequately characterized previously using B3LYP and cam-B3LYP functionals in studies of nitrobenzenes,^42,53^ nitrophenols,^44,59^ and nitroaromatic phototriggers.^25,38^ The integral equation formalization variant of the polarizable continuum model (IEFPCM) was used to describe implicit solvation in DMSO. These calculations were implemented in Gaussian 16.^60^ Molecular structures and orbitals were visualized using the Avogadro software.^61^

## 3 Results and Discussion

The dynamics of two nitroaromatic phototriggers, DMNB-Ser and NB-Tyr, that follow electronic excitation are investigated using two complementary transient absorption spectroscopy (TAS) techniques over a broad range of time scales. TEAS is primarily used to observe changes in excited-state absorption (ESA) profiles, whereas TVAS is used to monitor the recovery of ground-state bleach (GSB) features to provide information on the repopulation of the ground electronic states and hence deduce the efficiency of photorelease of serine or tyrosine. Overviews of the photochemistry observed in this study are shown in section 3.1. Section 3.2 considers subnanosecond dynamics in the singlet manifold, using TVAS data to identify prompt GSB recovery kinetics on short time scales. Triplet-state dynamics, the formation of metastable *aci*- isomers on the ground state, and deprotonation pathways are described in section 3.3. The behaviors of DMNB-Ser and NB-Tyr are summarized, compared, and contrasted in section 3.4 before the influence of mixed solvents on the observed behaviors is reported in section 3.5.

### 3.1 Overview of the Photochemical Pathways

**Figure 3:**
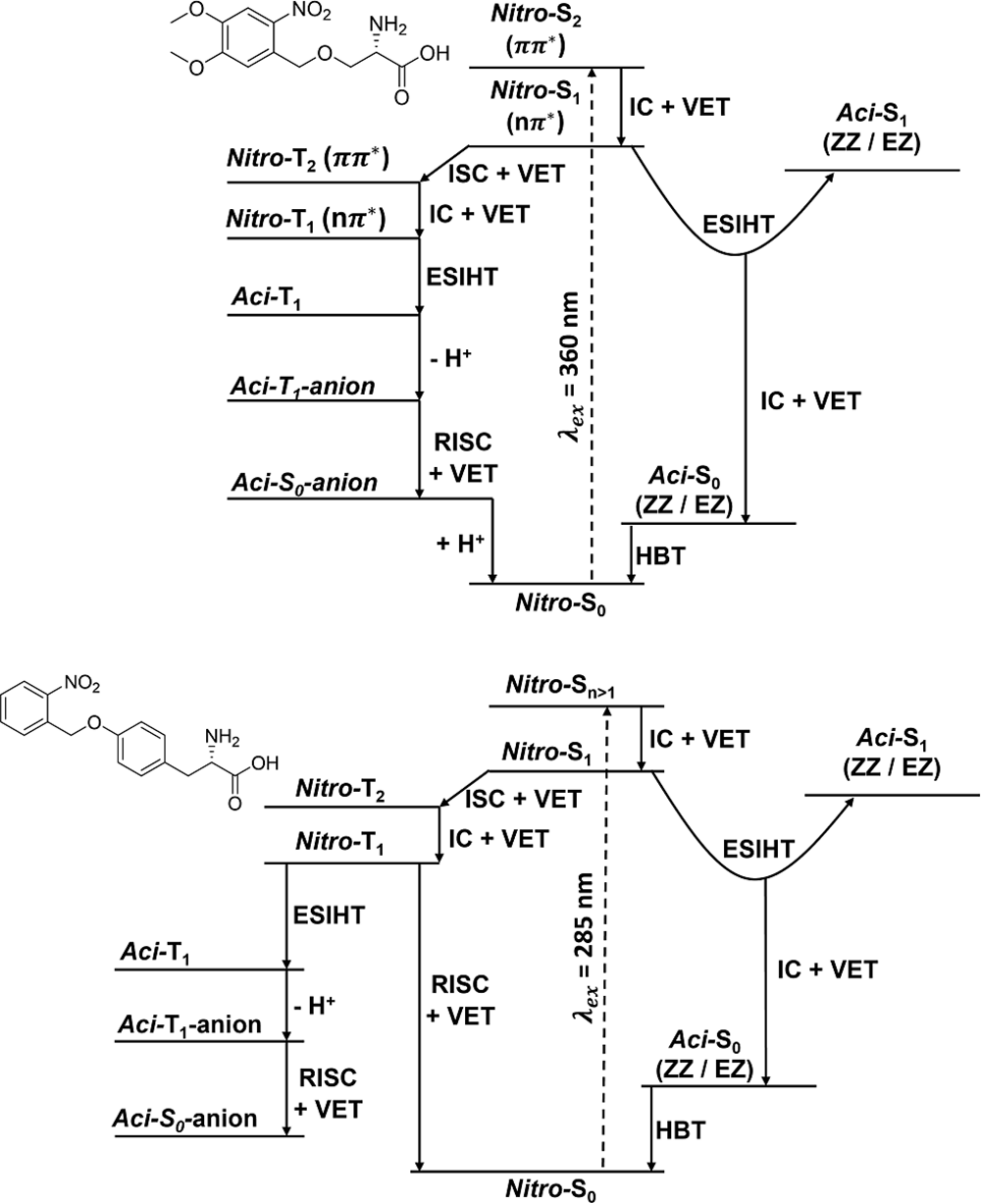
Photochemical pathways summarizing the proposed relaxation mechanisms for (top) DMNB-Ser and (bottom) NB-Tyr in DMSO solution following UV excitation. Dashed vertical arrows represent electronic excitation to populate excited singlet states, and solid arrows represent individual photophysical or photochemical processes. Internal energy is conserved in IC and ISC processes, but is lost by vibrational energy transfer (VET) to the surrounding solvent.

The photochemical pathways unraveled in the current work involve multiple competing and sequential steps. To facilitate their description, overviews of the proposed dynamics observed for DMNB-Ser and NB-Try using TAS methods are shown in Figure 3. The experimental and computational evidence in support of these mechanisms, and their associated time scales, is presented in sections 3.2 and 3.3.

Both DMNB-Ser and NB-Tyr contain nitroaromatic chromophores and hence show similar excited-state behavior in DMSO. The dynamics of these two studied PTs can be broadly divided into singlet-state photochemical pathways (section 3.2) and triplet-state photochemical pathways (section 3.3) following excitation by UV light. Characterization of the accessed excited states is guided by TDDFT calculations, the outcomes of which were compared with high-level theoretical calculations for nitrobenzene (NB) (section S2.2 of the Supporting Information).^39,41^ For DMNB-Ser the electronic characters of the excited states are explicitly labeled where appropriate, and initial photoexcitation is determined to be to the *nitro*-S_2_ (ππ*) state. However, for NB-Tyr, there is ambiguity from the TDDFT calculations about the precise electronic state(s) accessed on UV photoexcitation because of several close-lying singlet states, therefore, some state labels are omitted here. Hence, photoexcitation is shown in Figure 3 to be to an S_*n*_ state with *n* > 1. In both cases, rapid (sub-ps) internal conversion (IC) populates the *nitro*-S_1_ excited state.

Early time (*t* < 20 ps) TEAS measurements for each PT show the depopulation of *nitro*-S_1_ states, which we attribute to competitive ESIHT and ISC processes, with evidence from our combined TEAS and TVAS observations (sections 3.2 and 3.3) and the calculations of Mewes and Dreuw for *o*-nitrobenzyl acetate.^8^ The dynamics in the singlet state are dominated by an ESIHT pathway that describes the tautomerization of electronically excited PTs from the *nitro*-S_1_ isomer toward the more stable *aci*-S_1_ isomer. Our complementary TVAS measurements show approximately 70% GSB recovery on the order of 10 ps, which can be accounted for by efficient IC via conical intersections (CIs) along the ESIHT coordinate^8^ and subsequent hydrogen back-transfer (HBT) to reform the ground electronic state.

Within the triplet-state manifold, accessed via competitive ISC from the *nitro*-S_1_ state, the observed photochemical pathways differ somewhat between DMNB-Ser and NB-Tyr. Guided by our TEAS measurements and a second, slower component of parent-molecule ground-state recovery observed by TVAS, we propose that after populating *nitro*-T_1_ states, both PTs undergo ESIHT to their lower energy *aci*-T_1_ isomers, from which deprotonation occurs in solution to yield triplet *aci*- anion isomers. Spectroscopic evidence for anion formation and reasons for discounting other relaxation pathways are discussed in Section 3.3. For DMNB-Ser, the time scale for the second component of ground-state recovery (∼200 ns) can then be explained by RISC (or perhaps triplet quenching by dissolved oxygen) from *aci*-T_1_ anions to *aci*-S_0_ anions before reprotonation in the ground electronic state repopulates *nitro*-S_0_ directly. TVAS measurements for NB-Tyr reveal that the second component of partial GSB recovery occurs on a much faster time scale of 250 ps, which we attribute to direct *nitro*-T_1_ to *nitro*-S_0_ reverse intersystem crossing (RISC) based on the known photochemistry of other nitroaromatic compounds in solution.^41^

### 3.2 Singlet-State Dynamics

For TAS experiments, excitation wavelengths of 360 and 285 nm were selected for DMNB-Ser and NB-Tyr, respectively. Informed by UV–visible absorption spectra measured for solutions of each PT in DMSO-*d*_6_ (section S2.2 of the Supporting Information), these excitation wavelengths were selected to excite preferentially the low-energy edges of the first absorption bands, resulting in a population of low-lying electronically excited states with modest amounts of excess vibrational energy.

**Figure 4:**
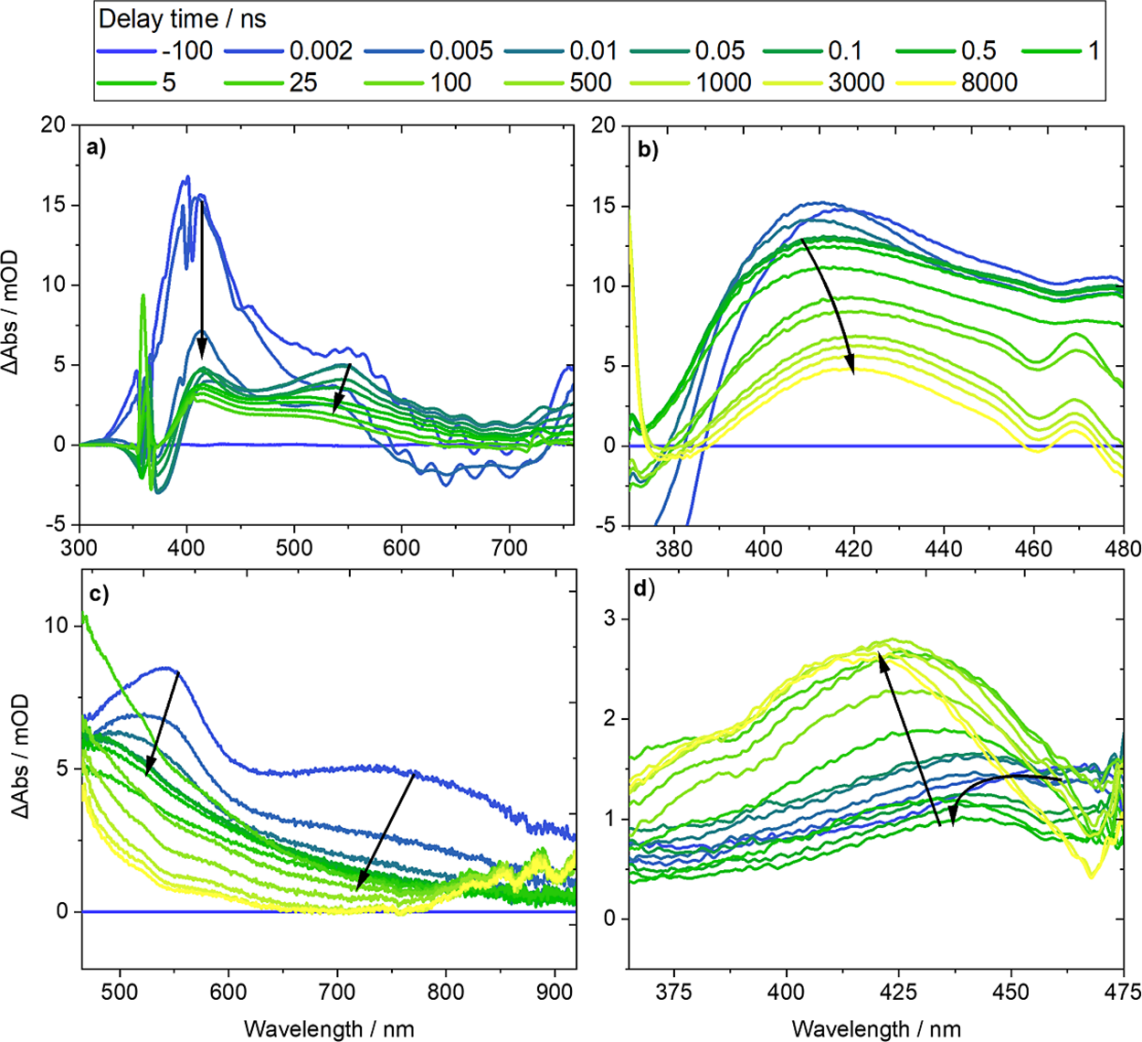
Transient electronic absorption spectra obtained for DMSO solutions of (a–c) DMNB-Ser excited using a 360 nm UV pump pulse, and (d) NB-Tyr excited using a 285 nm UV pump pulse. White-light continuum probe pulses were generated using (a) a CaF_2_window pumped by an 800 nm, 35 fs pulse, (b,d) a 4 mm sapphire window pumped by a 515 nm pulse, and (c) a 4 mm sapphire window pumped by a 1030 nm pulse to give different probe spectral ranges. Time delays after excitation were observed for (a) 200 fs–3.5 ns, and (b–d) 2 ps–8 μs. Spectra are colored to indicate the delay time of the white-light continuum probe pulse, and black arrows show the directions of changes of band intensities with time.

Transient electronic absorption spectra for DMNB-Ser and NB-Tyr in DMSO are presented in Figure 4. The TEAS measurements shown for DMNB-Ser correspond to the observation of early time dynamics with high temporal resolution (100 fs) over a broad spectral range (Figure 4a), as well as two measurements that observe a much broader range of time delays (1 ps–8 μs), albeit with lower time resolution and more limited spectral ranges (370–480 nm and 470–920 nm, respectively, Figure 4b,c, corresponding to the two WLC ranges from experiments at the Rutherford Appleton Laboratory’s LIFEtime facility). A single set of TEAS measurements is presented for NB-Tyr to illustrate changes over extended time delays (1 ps–8 μs) observed in the 370–475 nm probe region (Figure 4d).

Excitation of DMNB-Ser using a 360 nm laser pulse populates the *nitro-*S_2_ (ππ*) electronic state, resulting in a high-intensity ESA feature centered around 405 nm in TEAS measurements at short time delays (Figure 4a). This band rapidly decays on a time scale comparable to our instrument response function (IRF) of 110 fs, with commensurate growth of a broad ESA feature with two band maxima around 415 and 550 nm. This evolution of the ESA features is assigned to ultrafast internal conversion from the *nitro-*S_2_ (ππ*) state to the *nitro-*S_1_ (nπ*) state, which is optically inaccessible from the ground electronic state, with a time constant τ_IC_ ∼ 130 ± 10 fs obtained from kinetic fitting. Decay of a broad stimulated emission (SE) band centered around 670 nm, and assigned to the *nitro-*S_2_ (ππ*) state population, is also observed on this time scale. This SE decay is consistent with IC from an optically bright to a dark state. Our TEAS measurements do not resolve the subpicosecond dynamics for NB-Tyr that directly follow its excitation to a higher energy *nitro-*S_*n*_ state by absorption of 285 nm light. However, it is likely that similarly ultrafast IC occurs to populate the *nitro*-S_1_ state of NB-Tyr, from which the subsequent dynamics characterized here occur.

**Figure 5:**
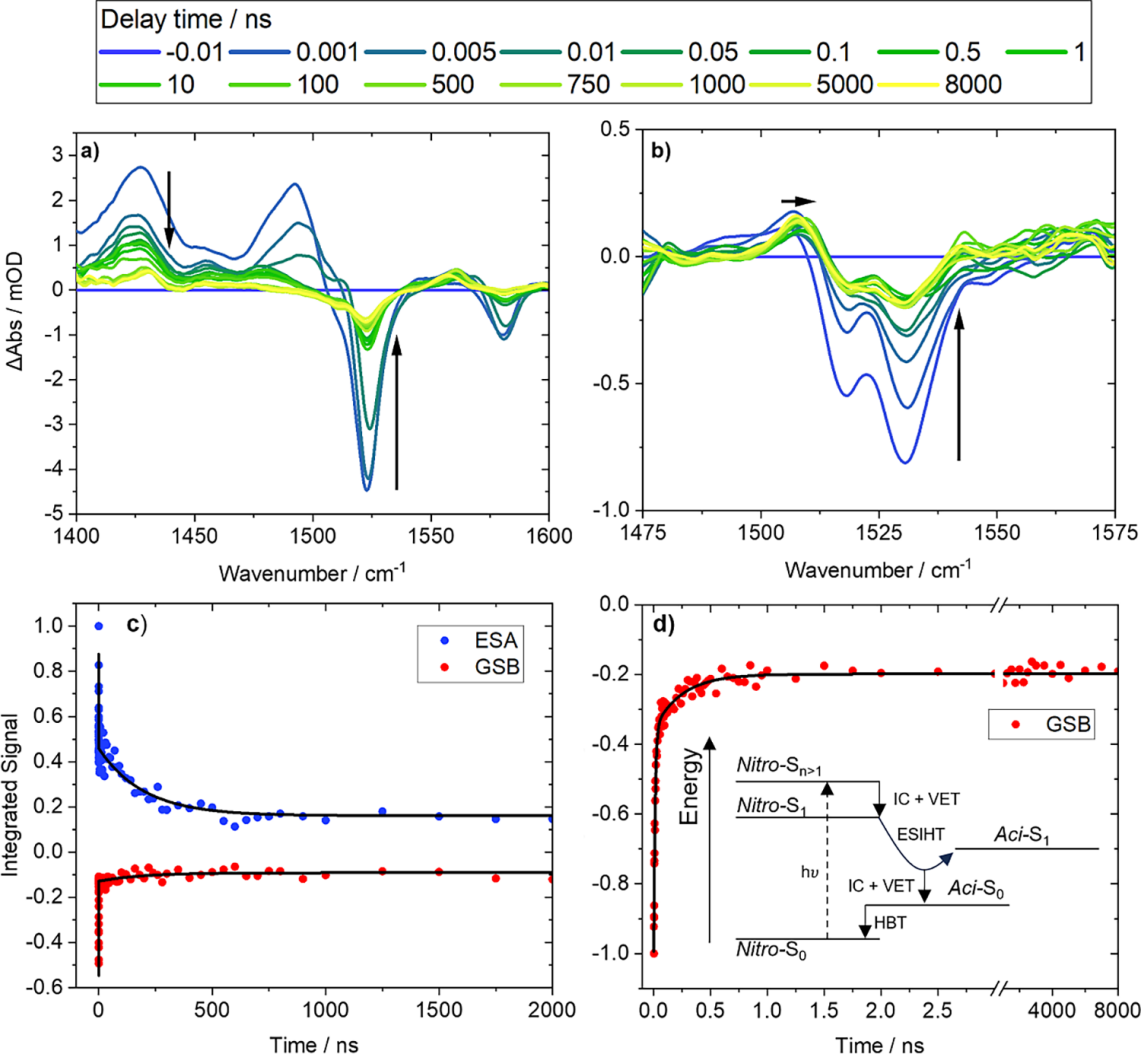
Transient vibrational absorption spectra obtained for time delays from 1 ps to 8 μs for DMSO-*d*_6_ solutions of (a) DMNB-Ser excited using a 360 nm UV pump pulse and (b) NB-Tyr excited using a 285 nm UV pump pulse. Spectra are colored to indicate the delay time of the broadband IR probe pulse. Also shown are kinetic traces for (c) DMNB-Ser, and (d) NB-Tyr determined from TVAS measurements in DMSO-*d*_6_. Solid lines are biexponential fits to data points (circles) that represent the integrated signals of basis functions used to model the evolution of the spectra over time. Time constants for TVAS measurements are presented in Tables 1 and 2. The inset in (d) is a Jablonski diagram showing a GSB recovery pathway for PTs in the singlet-state manifold.

Following ultrafast IC in DMNB-Ser, TEAS measurements report a hypsochromic shift in the 415 nm ESA band maximum to 410 nm (Figure 4a, b), as well as a decay of the positive band centered around 550 nm (Figure 4a, c). The shift of the former ESA band maximum is observed in the 300–750 nm probe region with a time constant of 2.46 ± 0.09 ps and in the 370–480 nm probe region with a time constant of 1.93 ± 0.03 ps. Each of these time constants corresponds to the same photophysical process, but they are extracted from different measurements. The decay of the ESA band centered around 550 nm is observed using the WLC probe spanning the 470 to 920 nm range and has a time constant of 3.0 ± 0.1 ps. TVAS spectroscopy of DMNB-Ser, as exemplified in Figure 5a, highlights a significant recovery in the GSB feature at 1525 cm^–1^ assigned to a NO_2_ asymmetric stretching mode coupled to a stretching mode of the DMNB ring in the S_0_ state (section S2.3 of the Supporting Information), with a comparable time scale of 7.65 ± 0.44 ps. This GSB recovery indicates repopulation of the *nitro-*S_0_ electronic ground state on sub-10 ps time scales, whereas the spectral changes observed via TEAS represent the depopulation of *nitro*-S_1_ (nπ*) in the same period. As the extracted time constants are similar, we interpret the shifting 415 nm band as evidence of *nitro*-S_1_ (nπ*) to *nitro-*T_2_ (ππ*) intersystem crossing (further discussed in Section 3.3), and the decay of the 550 nm S_1_ ESA band to arise from a combination of this ISC and indirect *nitro*-S_1_ (nπ*) to *nitro-*S_0_ IC via an ESIHT mechanism.^8^ The observation of GSB recovery using TVAS measurements excludes intramolecular vibrational energy redistribution (IVR) or VET to the solvent within the excited states of the PT molecules as possible assignments for the sub-20 ps time constants reported. Analysis of the fractional GSB intensity change shows that the ESIHT and S_1_ → S_0_ IC pathway accounts for 70% recovery of the DMNB-Ser photo-depleted ground-state population.

**Figure 6:**
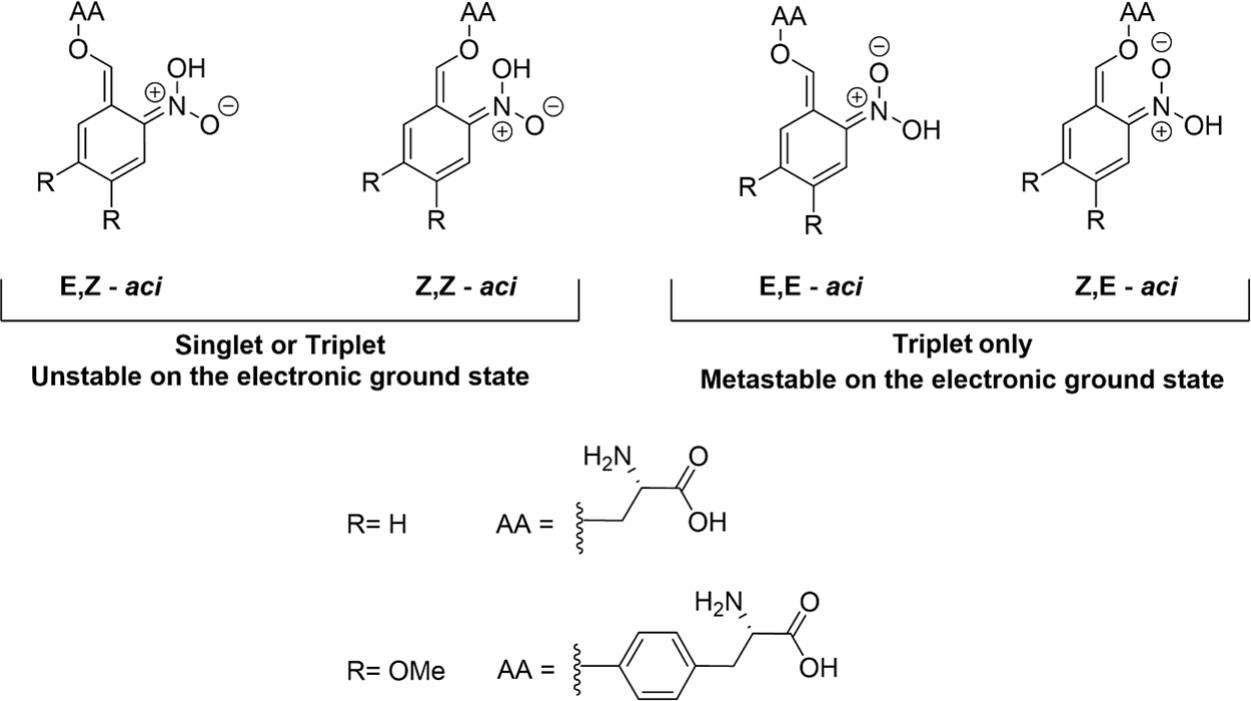
Possible *aci*- isomer distribution for general nitroaromatic phototriggers. The E,Z and Z,Z-*aci* isomers are formed directly from ESIHT. Singlet and triplet descriptions indicate the spin states that the isomers are accessible from (see main text). Stability on the electronic ground state refers to HBT.

As discussed in section 1, ESIHT can occur for photoexcited *ortho*-nitrobenzene chromophores to yield *aci-* isomers that are lower energy structures on the excited state potential energy surfaces (PESs) compared to their *nitro-* isomers.^8,55^ These *aci*- isomers are labeled according to the stereochemistry around the C=C and C=N double bonds formed by tautomerism, taking the form of X,Y- *aci-* where X and Y refer to the E/Z stereochemistry of the C=C and C=N bonds, respectively. In the singlet manifold, electronic rearrangement to form new double bonds during hydrogen transfer to the nearby oxygen atom occurs on shorter time scales than bond rotations. As a result, the C=N bond becomes conformationally locked in a single configuration, resulting in only Z stereochemistry. In contrast, because ESIHT can occur regardless of the geometry of the C–C bond, the stereochemistry of the C=C bond formed by ESIHT may be either E or Z. Therefore, two isomers are expected to form in the singlet manifold, E,Z-*aci-,* and Z,Z-*aci-* (Figure 6). Despite the greater energetic stability of *aci-*S_1_ isomers compared to *nitro-*S_1_ isomers, CIs accessible along the ESIHT coordinate are known to deactivate the excited state population via efficient IC to the ground electronic state, forming E,Z-*aci-*S_0_ and Z,Z-*aci-*S_0_ species.^8^ These isomers are unstable on the electronic ground state and subsequently undergo rapid HBT to reform the *nitro-*S_0_ state.

An alternative photophysical pathway for the depopulation of the *nitro-*S_1_ (nπ*) state is rapid ISC into the triplet-state manifold. After population of the *nitro*-T_1_ (nπ*) state, ESIHT can occur resulting in triplet *aci*- molecules that can freely convert between X, Y- *aci*- isomers. Subsequent relaxation to the ground electronic state yields four ground-state isomers that are either metastable or unstable to HBT, as shown in Figure 6. The triplet-state dynamics are discussed further in section 3.3.

Repopulation of the ground state via ESIHT, IC, and subsequent HBT is consistent with the time constants determined using TAS, and also with dynamics reported for other NB chromophores.^2,8,25,43,53^ TEAS measurements indicate that depopulation of the DMNB-Ser *nitro*-S_1_ (nπ*) state occurs over 2–3 ps via ESIHT and ISC pathways, which is supported by TVAS measurements that directly observe the repopulation of the S_0_ vibrational ground state with a time constant of 7.65 ± 0.44 ps. The GSB recovery time constant is slightly greater than that of the *nitro-*S_1_ (nπ*) depopulation time constant due to additional relaxation dynamics occurring as the population undergoes IC, HBT, and vibrational cooling in the ground electronic state.

Transient absorption spectra for NB-Tyr are presented in Figures 4d and 5b and show comparable early-time dynamics to DMNB-Ser. TVAS measurements show substantial recovery of the GSB band assigned to NO_2_ stretching vibrations with a time constant of 11.9 ± 0.6 ps, which is comparable to the 13.9 ± 1.2 ps time constant in TEAS measurements that describes the evolution of an ESA band centered around 470 nm into another band centered around 440 nm. As for DMNB-Ser, the change in ESA bands observed using TEAS of NB-Tyr is assigned to ISC from the *nitro*-S_1_ state into the triplet manifold. Recovery of the GSB feature on this time scale indicates that part of the *nitro*-S_1_ population instead relaxes to reform the ground-state *nitro*-S_0_ species, either directly via IC or indirectly via an ESIHT channel. Although CIs encountered along the ESIHT coordinate are known to be efficient deactivation pathways for *ortho*-nitrobenzyl compounds as discussed above, direct S_1_ to S_0_ IC channels have also been reported for nitrobenzene.^39,42,51^ Therefore, the possibility of direct IC via another relaxation coordinate (most likely associated with changes to the O–N–O bond angle and torsion of the NO_2_ group) should not be ignored.

The above analysis indicates that depopulation of the *nitro*-S_1_ state for both DMNB-Ser and NB-Tyr is described by both ISC and competitive ESIHT/IC photophysical processes. Furthermore, *nitro-*S_0_ repopulation characterized by GSB recovery occurs as a direct consequence of IC along the ESIHT coordinate in the singlet manifold^8^ and is therefore influenced by the rate of depopulation of the *nitro-*S_1_ state. For clarity hereafter, the time constant τ_S1_ will be used to describe the lifetime of the *nitro-*S_1_ state as this lifetime incorporates these competing deactivation processes, and it indirectly describes GSB recovery kinetics. Where appropriate, the repopulation of the ground state will be described separately.

### 3.3 Triplet-State Dynamics

Photoexcited nitrobenzene and other nitroaromatic compounds have been shown to undergo ISC on femtosecond to picosecond time scales,^42,43,62^ and a similar behavior is observed here for UV-excited DMNB-Ser and NB-Tyr from shifts of their S_1_ ESA bands to shorter wavelengths on the time scale of *nitro-*S_1_ depopulation (τ_S1_). According to El Sayed’s rules,^63^ ISC in DMNB-Ser will proceed via an intermediate *nitro*-T_2_ (ππ*) state to accommodate a change in orbital character from *nitro-*S_1_ (nπ*); this ISC will be followed by rapid *nitro*-T_2_ (ππ*) to *nitro*-T_1_ (nπ*) internal conversion. Assignment of the broad ESA band observed in our TEAS measurements, with a maximum of around 410 nm, to a triplet excited state, is supported by TAS measurements made for a series of 4,5-dimethoxynitrobenzyl acetate compounds.^55^ TA spectra for these structurally related compounds revealed a similar spectral profile to our observations for DMNB-Ser, to which Bley et al. assigned triplet character. Similar dynamics are observed for NB-Tyr on τ_S1_ time scales, with the evolution of an ESA band centered around 470 nm into a band centered around 440 nm attributed to ISC and subsequent IC to the *nitro-*T_1_ excited state. For both PTs, isosbestic points between the *nitro-*S_1_ and *nitro-*T_1_ ESA bands are not observed during the ISC process. We suggest that this is because population transfer proceeds via an intermediate *nitro*-T_2_ state and because the *nitro*-T_1_ state will be populated with excess vibrational energy that cools by VET on a time scale that is comparable to the few-ps ISC kinetics. In addition, some delay-time dependent spectral overlap may arise from hot ground-state absorption bands associated with internally excited S_0_ molecules competitively populated by IC from S_1_.

Following the ISC dynamics, a second component of S_0_ GSB recovery is observed in TVAS measurements for NB-Tyr with a time constant of 246 ± 32 ps. A comparable time constant of 346 ± 57 ps is independently observed in our TEAS measurements, corresponding to the decay in intensity of our assigned *nitro-*T_1_ ESA feature toward the baseline. Because a change in spin is required for this T_1_ → S_0_ relaxation, we refer to this time constant for reverse intersystem crossing as τ_RISC_. The second component of GSB recovery observed in NB-Tyr using TVAS is proposed to occur via a direct *nitro*-T_1_ to *nitro*-S_0_ RISC pathway, whereas shifts of ESA bands to shorter wavelengths in the TEA spectra (Figure 4d) on the same time scale as τ_RISC_ are attributed to ESIHT from the *nitro*-T_1_ state to the *aci*-T_1_ state (with ESA peaking at ∼430 nm for *aci*-T_1_ NB-Tyr). GSB recovery via a T_1_ – S_0_ triplet-singlet crossing was reported for photoexcited nitrobenzene,^41^ and is considered more likely in NB-Tyr than a radical-mediated pathway because homolytic bond cleavage in the triplet manifold would result in the formation of a radical triplet pair. This latter possibility cannot be fully discounted on the strength of our experimental evidence, but the radical triplet pair must then undergo a spin-flip to form a singlet radical pair before geminate recombination causes the observed GSB recovery. Inspection of the excited state *aci* structures in Figure 6 suggests the most plausible homolytic bond cleavage pathway to radical fragments is neutral H atom loss by O–H bond dissociation. After a spin change, H atom geminate recombination could reform the S_0_ molecules in their *nitro*- or *aci*- isomers. However, in a polar solvent like DMSO, we propose that heterolytic loss of an H^+^ ion, as discussed further below, will be preferred to homolytic loss of an H atom.

Unlike for the singlet excited states, ESIHT in the triplet manifold efficiently forms *aci-* isomers on the T_1_ potential energy surface, despite the presence of a crossing point between the T_1_ state and the S_0_ ground electronic state. The dynamics along the ESIHT coordinate can avoid ISC pathways at the triplet-singlet crossings which are mediated by the spin–orbit coupling. Decay of the *nitro*-T_1_ ESA band and incomplete GSB recovery are indicative of the competitive photophysical pathways for depopulation of the *nitro*-T_1_ state, highlighting that a fraction of the molecules does not repopulate the ground state and instead persists on the *aci*-T_1_ surface, most likely as four distinct isomers.

**Figure 7:**
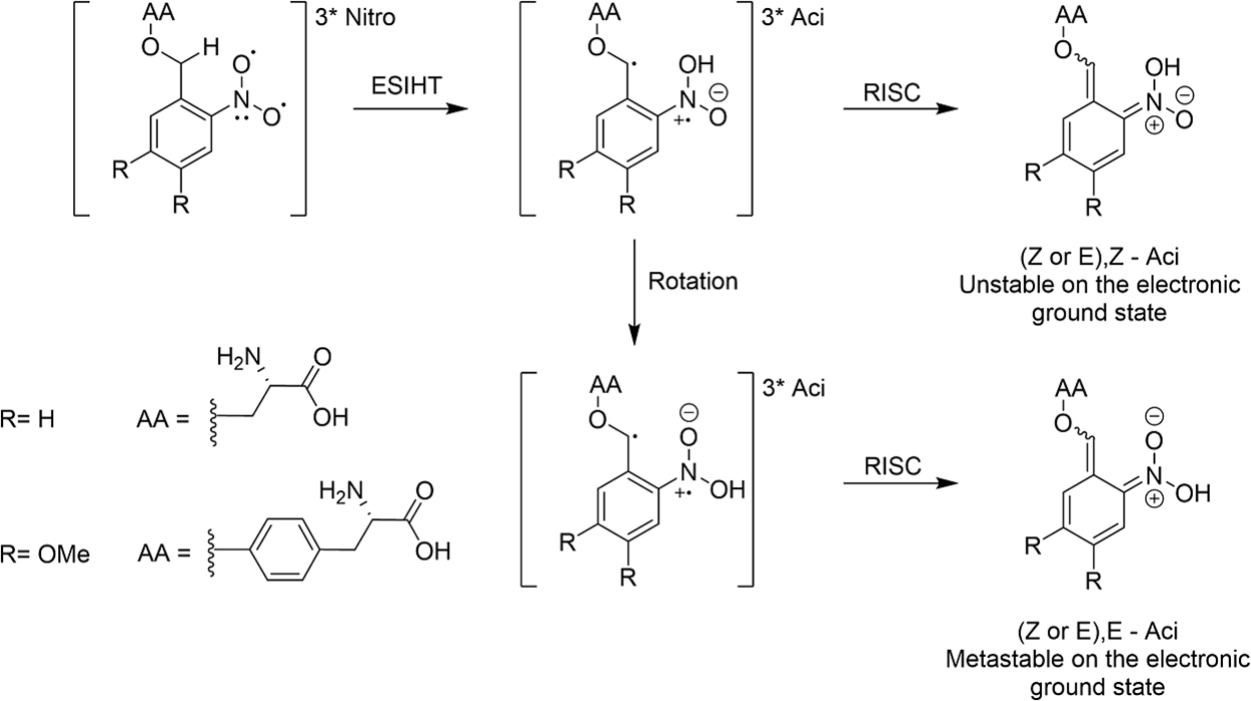
Triplet-state ESIHT and torsional dynamics in nitroaromatic phototriggers that result in four distinct *aci*- conformations.

As was discussed in section 3.2, ESIHT in the singlet manifold yields two *aci-* isomers that are unstable on the electronic ground state with respect to HBT. The instability arises from the conformational restriction of the C=N bond to a Z configuration, resulting in a geometry for which HBT can occur, therefore facilitating GSB recovery. However, consideration of the radical character of the triplet states suggests that there can be unrestricted rotation about the C–N bond after the initial hydrogen transfer. These torsional dynamics allow E configurations about the C=N bond to be accessed, and therefore, a total of four *aci-* isomers can potentially form in S_0_ following RISC, as shown in Figure 7. Furthermore, the Z,E and E,E-*aci* isomers arising from these triplet-state dynamics will be metastable on the electronic ground state with respect to HBT because the transferred hydrogen atom is spatially separated from the alkene moiety.

Using our TVAS measurements, the percentage of GSB recovery can be quantified. From this analysis, the fraction of electronically excited molecules that form *aci*- isomers that are metastable to HBT can be deduced, assuming that any isomers that are unstable to HBT contribute to the recovery of the ground electronic state *nitro-* form on the time scale of our measurements. In DMSO, approximately 13% of the photoexcited molecules relax to metastable *aci*- isomers for DMNB-Ser, compared to 20% for NB-Tyr. Quantum yields of photodeprotection reported for ONB PTs are in the range of 10–20%,^8^ which is consistent with our estimates of the formation of metastable *aci*- isomers for the two nitroaromatic PTs studied here. Full details of these estimates are provided in the Supporting Information, section S3.6. We note that there is no evidence from our TEAS or TVAS measurements of alternative decomposition pathways involving nitrous acid (HONO) elimination. Direct HONO elimination from a T_1_ or S_1_*aci* species should yield triplet or singlet biradical or carbene cofragments, which are also not observed.

At time delays on the order of nanoseconds, the TEA spectra for NB-Tyr are dominated by a high-intensity, broad ESA band centered around 425 nm that grows with a time constant of 10.2 ± 0.5 ns. A comparable time constant of 9.4 ± 0.7 ns can be extracted from TEAS measurements for DMNB-Ser, corresponding to a shift in the band maximum and reduction of the intensity of the feature assigned to absorption from either *nitro*-T_1_ or *aci*-T_1_ DMNB-Ser. The resultant ESA feature is assigned to *aci-* anions formed via deprotonation at the *nitro-* moiety following ESIHT on the T_1_ PESs, with a deprotonation time constant τ_D_. These *aci*- anions must initially form in their T_1_ state, but we cannot distinguish T_1_ from S_0_ anions in the assignment of the 425 nm band. The assignment of these features is informed by TDDFT calculations for *aci*-S_0_ anions (section S2.4 of the Supporting Information) and studies of comparable molecules.^55,64^ TVAS measurements of NB-Tyr (Figure 5b) show the same time constant, τ_D_ = 11.6 ± 5.2 ns, corresponding to a shift in the ESA band centered around 1510 cm^–1^ to smaller wavenumber by approximately 5 cm^–1^. The change in the observed vibrational wavenumber is again attributed to the deprotonation of *aci*-T_1_ isomers to form *aci*- anions.

In the case of DMNB-Ser, our TVAS measurements show no evidence of a second GSB recovery component on the ∼250 ps time scale for τ_RISC_ observed for NB-Tyr. As repopulation of the ground electronic state is not observed on the order of picoseconds, direct (or radical mediated) *nitro*-T_1_ to *nitro*-S_0_ RISC is discounted as a competitive deactivation route for DMNB-Ser. Instead, shifts to shorter wavelength in the ESA bands suggest that the excited state population undergoes ESIHT to the *aci*- form on the T_1_ PES and subsequent deprotonation to yield *aci*-T_1_ anions, as is proposed to occur in NB-Tyr. Deprotonation from the *aci-*T_1_ state should yield anions in both isomer configurations, but this deprotonation at the N(O)OH site removes the necessity of conformer labeling about the C=N bond because the anionic forms are equivalent. While deprotonation of DMNB-Ser might occur from the ground *aci*-S_0_ electronic state following RISC from the *aci*-T_1_ state, studies of various nitrophenol molecules performed in our laboratory have shown that deprotonation occurs within a few nanoseconds from their triplet excited states. Hence, we propose that deprotonation also occurs from the triplet-state manifold for the nitroaromatic PTs, which are therefore behaving as photoacids, albeit after rapid ESIHT.^64^

Nevertheless, TVAS measurements for DMNB-Ser do reveal a second, slow component of GSB recovery, with an estimated time constant of 197 ± 15 ns. This time scale is comparable to τ_Iso_ = 440 ± 40 ns observed using the short wavelength WLC probe in TEAS measurements of photoexcited DMNB-Ser. Because the associated absorption band is more pronounced in TEAS measurements, we consider the τ_Iso_ = 440 ± 40 ns time constant to be more reliable. Partial GSB recovery indicates that over later times, there is a mechanism for repopulation of the *nitro*-S_0_ state from the anionic species. Additionally, Figure 4b highlights the evolution of the ESA feature assigned to a DMNB-Ser anion toward a band with a peak maximum of 419 nm. The final band position observed using TEAS for DMNB-Ser has the same central wavelength as that of the final band observed for NB-Tyr. Assuming deprotonation in excited-state DMNB-Ser yields *aci-*T_1_ anions as discussed earlier, we postulate that RISC accesses *aci-*S_0_ anions in both E and Z conformations with respect to the C=C bond. From the *aci*- ground electronic S_0_ state, DMNB-Ser anions can either protonate to reform the *nitro*-S_0_ species directly, bypassing the HBT that is required for deactivation pathways in the singlet-state manifold, or undergo isomerization to yield Z-*aci*-anions, as is discussed below for NB-Tyr. The 200–440 ns τ_Iso_ time constant therefore is proposed to correspond to a relaxation of the excited *aci*-T_1_-anion population into the Z-*aci*-S_0_ and *nitro*-S_0_ states, and it accounts for the second component of GSB recovery, and the shift of the ESA band position over late-times.

Late-time (*t* > 1 μs) TEAS measurements for NB-Tyr reveal a shift in the ESA band maximum by approximately 12 nm toward a shorter wavelength on a time scale of a few microseconds. Our TDDFT calculations for the electronic ground state of the anion (section S2.4 of the Supporting Information) indicate that the vertical excitation energy for Z-*aci*-anions with respect to the C=C bond is higher than that for E-*aci*-anions by an amount corresponding to a 10 nm wavelength shift. Because of this close correspondence, the evolution of the spectra is proposed to be due to isomerization from E-*aci*-anions to Z-*aci*-anions, in this instance, with a time constant τ_Iso_ = 1.01 ± 0.16 μs.

### 3.4 Summary of the Photochemical Dynamics in DMSO

Schematic overviews of the proposed photochemical pathways observed for DMNB-Ser and NB-Try using transient absorption spectroscopies are shown in Figure 3 (section 3.1), and summaries of the time constants deduced for the various steps are presented in Tables 1 and 2.

**Table 1:** Time Constants for DMNB-Ser Dynamics Measured Using TEAS and TVAS in Solutions of DMSO or DMSO-*d*_6_

probe type	time constants			
	τ_IC_/ps^c^	τ_S1_/ps	τ_D_/ns	τ_Iso_/ns
CaF_2_ WLC (300 – 750 nm)	IRF^a^	2.46 ± 0.09		
WLC (370 – 480 nm)		1.93 ± 0.03	9.4 ± 0.7	440 ± 40
WLC (470 – 920 nm)		3.0 ± 0.1		
IR^b^		7.65 ± 0.44		

a Measurement is limited by the IRF of the laser system.

b For TVAS measurements, time constants directly report on GSB recovery dynamics.

c τ_IC_ refers to IC between the *nitro*-S_2_ (ππ*) and *nitro*-S_1_(nπ*) states, as described in sections 3.1 and 3.2.

**Table 2:** Time Constants for NB-Tyr Dynamics Measured Using TEAS and TVAS in Solutions of DMSO or DMSO-*d*_6_

probe type	time constants			
	τ_S1_/ps	τ_RISC_/ps	τ_D_/ns	τ_Iso_/ns
WLC (370 – 480 nm)	13.9 ± 1.2	346 ± 57	10.2 ± 1.5	1010 ± 160
IR^a^	11.9 ± 0.6	246 ± 32		

a For TVAS measurements, time constants directly report on GSB recovery dynamics.

Examination of the overall dynamics and associated time constants identifies many similarities between the two studied PTs. Both species absorb UV light through π* ← π transitions of the ONB chromophore common to both molecules, but modification with electron-donating methoxy substituents shifts the absorption to a longer wavelength. Longer excitation wavelengths for DMNB-Ser than for NB-Tyr are significant when considering applications of PTs, for example, in biological studies where lower-energy excitation might reduce damage to UV-sensitive samples.

Concerning the photochemical dynamics, relaxation mechanisms in the singlet manifold of electronic states are comparable for the two species: IC from the S_1_ state to the S_0_ state occurs efficiently via a conical intersection located along the S_1_ ESIHT coordinate,^8^ accounting for between 60 and 75% of the *nitro*-S_1_ state population relaxation in both phototriggers, before subsequent HBT repopulates the ground-state *nitro* species. A more involved route starting with ISC from *nitro*-S_1_ to populate excited triplet states also results in the recovery of the *nitro*-S_0_ ground state. This behavior is observed in other ONB derivatives,^8,55^ and therefore appears to be fundamental to the chromophore. Nevertheless, there are some discrepancies between the measured time constants that differentiate the photochemistry of NB-Tyr from that of DMNB-Ser. Considering depopulation of the *nitro*-S_1_ state, the S_1_ lifetime τ_S1_ is significantly shorter for DMNB-Ser compared to NB-Tyr, which is representative of either more efficient ISC, or more efficient ESIHT in the former molecule. A possible explanation stems from the methoxy substituents exclusive to DMNB-Ser having an electron-donating effect toward the ONB chromophore. If greater electronic density on the DMNB moiety perturbs the relative energies of the electronically excited states, then such changes could facilitate ESIHT and/or ISC, with a consequent reduction of τ_S1_ compared to the unsubstituted PT analogue.

Triplet-state dynamics following ISC show more variation between the two PTs. Both species undergo rapid ISC from the *nitro*-S_1_ state, then IC from the *nitro-*T_2_ state to the *nitro*-T_1_ state. However, from this common point in the photochemical pathways, we observe direct (or radical-pair mediated) *nitro*-T_1_ to *nitro*-S_0_ RISC with a time constant of τ_RISC_ = 246 ± 32 ps for NB-Tyr that is not evident for DMNB-Ser. In contrast, for DMNB-Ser, a GSB recovery component indicating a return to the *nitro*-S_0_ species develops on nanosecond time scales. This slow pathway is assigned to reprotonation of *aci*-S_0_-anions, themselves formed by the deprotonation of photoacidic *aci*-T_1_ molecules and relaxation of the resulting *aci*-T_1_ anions. Both studied nitroaromatic PTs are observed to undergo *nitro*-T_1_ to *aci*-T_1_ ESIHT, and subsequent deprotonation with comparable time constants, τ_D_≈ 10 ns. Analysis of the spectra at late times suggests that the same anionic intermediate (Z-*aci-*S_0_) forms for both species. Regardless of the exact route taken to reach the Z-*aci*-S_0_ species, further chemistry resulting in bond cleavage must occur from the same states in NB-Tyr and DMNB-Ser if these molecules are to act as phototriggers.

The previously discussed mechanism of photodeprotection in *ortho*- nitrobenzene PTs invoked singlet (S_0_) *aci-* isomers as key intermediates in the decaging process to release active molecules (here serine or tyrosine) and an *ortho*-nitroso coproduct.^38,55^ From the *aci-* intermediate, intramolecular nucleophilic attack at the alkene moiety by a *nitro*- oxygen atom forms a five-membered ring. N–OH deprotonation then facilitates a ring-opening process, with the elimination of the active moiety as an anion. The final intermediates observed in our TAS experiments are posited here to be *Z-aci-*S_0_ anions, which we suggest are primed to initiate this photodeprotection, consistent with the proposed mechanisms. Because the anionic intermediates are deprotonated at the *nitro-* group, either oxygen atom can attack the alkene moiety in the cyclization step. In contrast, from a neutral *aci-* intermediate, the stereochemistry about the C=N bond determines whether the unsubstituted oxygen atom is correctly orientated for cyclization. After a reaction involving a neutral intermediate, deprotonation is necessary before the ring opening of the cyclic intermediate yields photoproducts, whereas for the *Z-aci-*S_0_ anions, this is not the case. The formation of *Z-aci-*S_0_ anions by the photochemical mechanisms proposed in Figure 3, and substantiated by our transient absorption spectroscopy measurements, is therefore consistent with the current understanding of photodeprotection mechanism of nitroaromatic phototriggers.

### 3.5 Photochemical Dynamics of NB-Tyr and DMNB-Ser in Mixed Solvents

**Figure 8:**
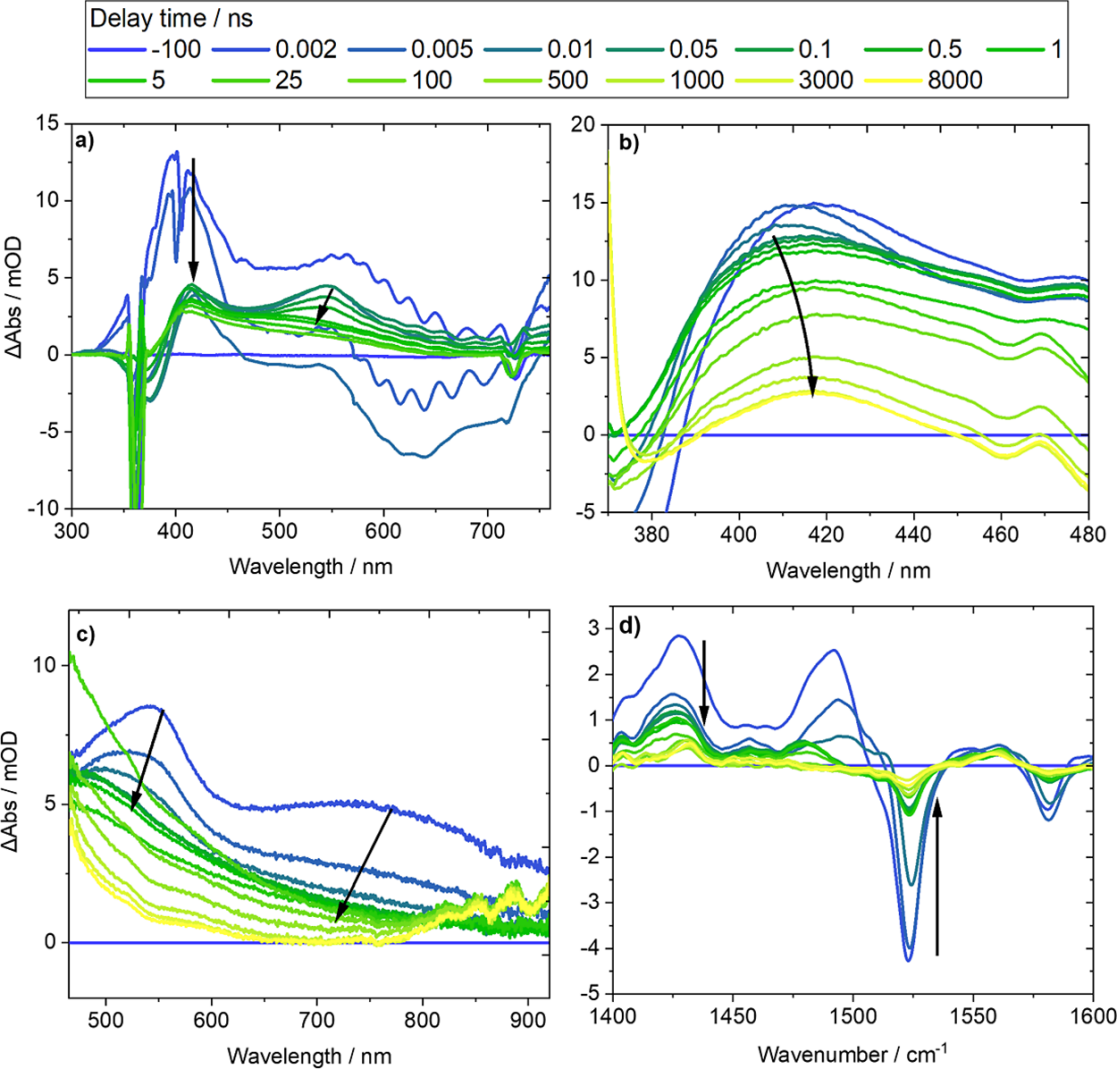
(a–c) Transient electronic absorption spectra and (d) transient vibrational absorption spectra for solutions of DMNB-Ser in mixed solutions of DMSO/DMSO-*d*_6_ (10 mL) and H_2_O/D_2_O (1 mL) excited using 360 nm light. Spectra are colored to indicate the delay-time of (a–c) the white-light continuum and (d) the broadband IR probe pulses. Black arrows show the directions of changes of band intensities with time. Time delays after excitation were observed for (a) 200 fs–3.5 ns, and (b–d) 2 ps–8 μs.

To explore further the influence of the environment on the observed photochemical pathways, mixed DMSO/water solutions were prepared by the addition of 1 or 2 mL of H_2_O to 10 mL of DMSO (or of D_2_O to DMSO-*d*_6_). The addition of water to DMSO gave mixed solvents that were approximately 9 and 17% water by volume for DMSO/H_2_O (1 mL) and DMSO/H_2_O (2 mL) solutions respectively. Transient absorption spectra of DMNB-Ser in DMSO/H_2_O (1 mL) are shown in Figure 8, and a complete list of time constants obtained from our analysis of these spectra is shown in Table 3. Discussion in this section will focus on DMNB-Ser photochemistry, with data for NB-Tyr available in sections S3.4 and S3.5 of the Supporting Information.

**Table 3:** Time Constants for DMNB-Ser Dynamics Measured Using TEAS in Mixed DMSO/H_2_O Solutions and TVAS in Mixed DMSO-*d*_6_/D_2_O Solutions

probe type	*V*_water_/mL	time constants				
		τ_IC_/ps^d^	τ_S1_/ps^a^		τ_D_/ns	τ_Iso_/ns
CaF_2_ WLC (300 – 750 nm)	0	IRF^b^	2.46 ± 0.09			
	1	IRF^b^	2.11 ± 0.06	35 ± 6		
	2	IRF^b^	1.70 ± 0.06	35 ± 5		
WLC (370 – 480 nm)	0		1.93 ± 0.03		9.4 ± 0.7	440 ± 40
	1		1.95	10.1 ± 1.2	5.0 ± 0.3	489 ± 17
	2		1.95	7.2 ± 0.4	5.2 ± 1.1	377 ± 14
WLC (470 – 920 nm)	0		3.0 ± 0.1			
	1		2.4 ± 0.2	10.5 ± 3.1		
	2		2.1 ± 0.3	7.9 ± 1.7		
IR^c^	0		7.7 ± 0.4			
	1		5.9 ± 0.4			
	2		4.9 ± 0.4			

a In mixed DMSO/water solutions, the S_1_ population decay has two exponential components.

b Measurement is limited by the IRF of the laser system.

c For TVAS measurements, time constants directly report on GSB recovery dynamics.

d τ_IC_ refers to IC between the *nitro*-S_2_ (ππ*) and *nitro*-S_1_ (nπ*) states, as described in sections 3.1 and 3.2.

The time constants summarized in Table 3 show no observable change in the value of τ_IC_ on the addition of water (9–17% by volume) to the DMSO solutions of DMNB-Ser. The rate of ultrafast IC from the optically accessible *nitro*-S_2_ (ππ*) to *nitro*-S_1_ (nπ*) occurs on the order of our IRF in all cases, regardless of the makeup of the solvent.

The addition of water to the DMSO solutions of the two PTs will perturb the relative energies of the excited states due to their different electronic characters; we expect the *nitro*-T_2_ (ππ*) state to be stabilized by protic interactions with water relative to its energy in DMSO, whereas the *nitro*-S_1_ (nπ*) state will be destabilized.^65−67^ Consequently, the energy gap for ISC (Δ*E*_ISC_) should increase in the presence of water, resulting in a decrease in the rate of ISC as the states diverge. Analysis of TEAS measurements for DMSO/H_2_O (1 mL) and DMSO/H_2_O (2 mL) solutions in all WLC probe regions (Figure 8) requires biexponential functions to describe the depopulation of the *nitro*-S_1_ (nπ*) excited state. The time constants extracted from these fits are reported in Table 3.

In the mixed solutions, there are two components to the *nitro*-S_1_ (nπ*) lifetimes for DMNB-Ser, a sub-3 ps component, and a longer (7–35 ps) component. For spectra measured using the 370–480 nm WLC probe region (Figure 8b) in mixed solutions, the *nitro*-S_1_ (nπ*) lifetime has a 1.95 ps component, and a longer (10.1 or 7.2 ps) component. However, in neat DMSO the *nitro*-S_1_ (nπ*) lifetime is well described by a single time component only, with τ_S1_ = 1.93 ± 0.03 ps. In the mixed solvents, the 1.95 ps decay component can therefore be attributed to DMNB-Ser molecules solvated exclusively by DMSO. The relative amplitudes for the two biexponential fitting components are shown in the Supporting Information S3.3 and reveal that the longer time components account for approximately 7 and 16% of the total fitting amplitudes in DMSO/H_2_O (1 mL) and (2 mL) solutions, respectively. Given that the composition of the mixed solvents is 9 and 17% water by volume, we propose that the additional time components observed for τ_S1_ in mixed solvents arise from interactions of DMNB-Ser with water molecules, perturbing the relative energies of the *nitro*-S_1_ (nπ*) and *nitro*-T_2_ (ππ*) states as described above. Consequently, a fraction of the excited state population, proportional to the percentage by volume of water in the solvent, undergoes slower ISC. The same arguments apply to τ_S1_ time constants measured using 300–750 nm and 470–920 nm WLC probe regions, where the sub-3 ps time constant decreases slightly from pure DMSO to mixed solvents, but a second, larger time constant is identified in the mixed solvents and is attributed to the interaction of DMNB-Ser with water.

**Figure 9:**
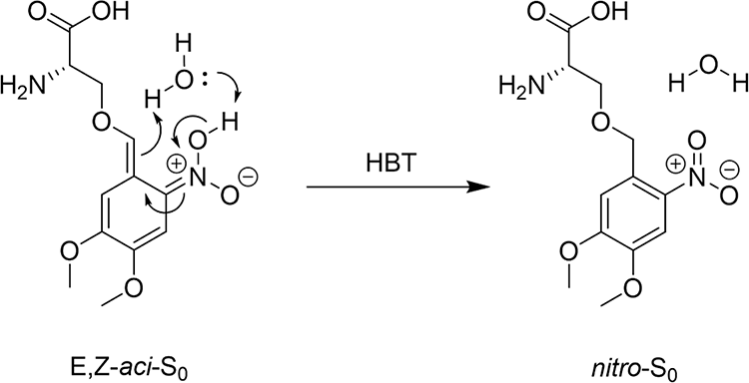
Proposed mechanism of water-assisted HBT for DMNB-Ser in the S_0_ state.

Unlike the WLC probes which report on excited-state populations, the IR probe in our TVAS measurements observes GSB recovery both promptly via singlet ESIHT, IC, and HBT, and over longer delays via indirect routes following ISC. The dependence of the GSB recovery lifetimes on the volume of water added to DMSO in the mixed solvent, shown in Table 3 as time constants determined using an IR probe, indicates that the ESIHT or HBT steps become faster with the addition of water. One possible role for the water molecules is to facilitate HBT processes that recover the ground electronic state, therefore reducing observed GSB recovery lifetimes. In the singlet manifold, IC along the ESIHT coordinate populates E, Z-*aci*-S_0_, and Z,Z-*aci*-S_0_ states which subsequently undergo HBT to reform the *nitro*-S_0_ state. In mixed DMSO/H_2_O solutions, the presence of water might catalyze the HBT process, either actively by behaving as a molecular wire to facilitate hydrogen atom transfer or as a source of protons in solution. A proposed mechanism is shown in Figure 9 where water behaves as a molecular wire in a concerted hydrogen exchange reaction.

The value of τ_D_ decreases with the addition of water, as shown in Table 3, suggesting that H_2_O facilitates deprotonation of excited-state *aci*-T_1_ DMNB-Ser in solution. Kinetics extracted from TVAS measurements indicate that there is a trend of increasing τ_Iso_ values with the addition of water. However, kinetics observed using our WLC probes do not exhibit an obvious correlation between solvent composition and τ_Iso_.

An increase in τ_Iso_ with the addition of water means slower recovery of the ground-state *nitro*-S_0_ population, as evidenced by slower decay of the GSB intensity. Therefore, according to our overall picture of the photochemical relaxation, either the RISC between the T_1_ and S_0_ states of the *aci* anions or reprotonation of the S_0_*aci* anions must be slower. The latter option appears unlikely in a solution containing more protic solvent molecules.

## 4 Conclusions

Transient electronic and transient vibrational absorption spectroscopy techniques have been used to explore the photochemistry of two UV-excited, ortho-substituted nitroaromatic phototriggered compounds, denoted here as NB-Tyr and DMNB-Ser, which are designed to release the amino acids tyrosine and serine. Measurements in DMSO solution ranging from subpicosecond to microsecond delay times reveal two primary photochemical pathways that are accessed either by excited state intramolecular hydrogen transfer or intersystem crossing from the electronically excited *nitro*-S_1_ state. Within the singlet-state manifold, efficient *nitro*-S_1_ to *aci*-S_0_ internal conversion on the order of 2–15 ps is facilitated by a conical intersection accessed along the ESIHT coordinate. Metastable *aci*-S_0_ isomers are then observed to repopulate the stable *nitro*-S_0_ ground state on the order of 5–15 ps by hydrogen back-transfer. Alternatively, competitive ISC from the *nitro*-S_1_ state to the *nitro*-T_2_ state accesses a more complex photochemical pathway, with subtle differences observed between NB-Tyr and DMNB-Ser. Following ultrafast *nitro*-T_2_ to *nitro*-T_1_ IC, evidence from our transient absorption spectroscopy suggests that the NB-Tyr population may undergo direct *nitro*-T_1_ to *nitro*-S_0_ reverse intersystem crossing with a time constant τ_RISC_ = 346 ± 57 ps in DMSO, or competitive ESIHT within the triplet manifold to yield *aci*-T_1_ isomers on the same time scale. For DMNB-Ser, direct *nitro*-T_1_ to *nitro*-S_0_ RISC is not observed; instead, the population all appears to undergo ESIHT from the *nitro*-T_1_ state. The growth of a spectroscopic feature assigned to the anionic *aci*- form suggests deprotonation of *aci*-T_1_ isomers with a time constant of τ_D_≈ 10 ns for both species in DMSO solutions. Modification of the *ortho*-nitrobenzyl-moiety therefore does not change the rate of deprotonation from the T_1_ state. The newly formed *aci*-T_1_ anions are argued to undergo RISC to produce *aci*-S_0_ anions, and a significant component of parent-molecule recovery for DMNB-Ser is observed over hundreds of nanoseconds that we attribute to the reprotonation of the ground-state anions.

The incomplete recovery of ground-state bleach features in TVAS measurements for both phototriggers is attributed to the persistence of Z-*aci*-anions. Given that Z-*aci*-anions are observed at our maximum time delay (8 μs), we propose that this species is a precursor to covalent bond cleavage which occurs on the order of milliseconds to liberate the amino acids serine and tyrosine.^55^ If Z-*aci*-anions must be formed prior to cleavage, and deprotonation occurs exclusively within the triplet manifold, it follows that ISC is a requirement for cleavage to occur in this class of ortho-substituted nitroaromatic PTs. Furthermore, if deactivation of the *nitro*-S_1_ state is dependent primarily upon competitive singlet ESIHT and triplet ISC pathways, then the maximum quantum yield of photoproduct formation (Φ_P_), corresponding to the fraction of initially excited species that do not reform the ground state parent PT molecule on our time scales, will be determined by the quantum yield for triplet formation (Φ_T_, Supporting Information S3.6). For pure DMSO solutions, Φ_T_ (NB-Tyr) is greater than Φ_T_ (DMNB-Ser), despite NB-Tyr having a significantly larger S_1_-state lifetime (τ_S1_). Estimates of the upper limits for Φ_P_ from TVAS measurements (S3.6) are Φ_P_ (NB-Tyr) = 20%, compared to Φ_P_ (DMNB-Ser) = 13%. Overall, these data suggest that NB-Tyr is a more efficient PT regarding amino acid release compared to DMNB-Ser, which may be due to changes in the electronic structure caused by methoxy ring-substituents in DMNB-Ser, or because of the shorter UV photoexcitation wavelength of NB-Tyr compared to DMNB-Ser. Comparisons between the PTs investigated here also show that the efficiency of photoinduced bond cleavage is determined by the relative efficiencies of ESIHT and ISC, rather than the lifetime of the *nitro*-S_1_ state.

TAS measurements conducted in mixed DMSO/H_2_O solvents have demonstrated that excited state photochemical behavior is sensitive to the solvation environment. Specifically, the addition of a protic solvent to DMNB-Ser in DMSO yields a second, longer S_1_-state lifetime component that arises due to interactions of a statistical fraction of the solute PT molecules with the minority of water molecules in the mixed solution. By stabilizing the *nitro*-T_2_ (ππ*) state relative to the *nitro*-S_1_ (nπ*) state in DMNB-Ser, the effect of water is to decrease the rate of ISC relative to ESIHT, decreasing Φ_T_ and therefore Φ_P_ compared to solvation in neat DMSO. A faster deprotonation (i.e., a shorter lifetime for deprotonation from the *aci*-T_1_ state, τ_D_) is observed using TEAS in mixed solvents, however, the rate of deprotonation has a negligible effect on the efficiency of photoinduced bond cleavage because the *aci*-T_1_ state is not a branching point in the excited state photochemical pathways. These observations demonstrate how transient absorption spectroscopy over femtosecond to microsecond time scales provides a comprehensive picture of competing photochemical pathways in the action of example nitroaromatic phototriggered compounds, although the final step of amino acid release remains too slow to be observed by the methods used here.

## Data Availability

Data are available at the University of Bristol data repository, data.bris, at https://doi.org/10.5523/bris.1azgkwaz40zsn1ydofuw4q5mda.

## Supporting Information Available

Additional details of spectroscopic methods, steady-state UV–visible absorption spectroscopy and FTIR spectroscopy for PTs in DMSO, DFT frequency calculations, TDDFT calculations, NTO projections for DMNB-Ser, spectral decomposition of TEA and TVA data in DMSO, TEAS and TVAS data for NB-Tyr, time constants for NB-Tyr, calculations for triplet quantum yield, branching yield, and quantum yield of photoproduct formation (PDF)
