# Supplementary material for: Femtosecond to Microsecond Observation of Photochemical Pathways in Nitroaromatic Phototriggers Using Transient Absorption Spectroscopy
Source: J Phys Chem A. 2024 Jul 11;128(29):5892–905. doi: 10.1021/acs.jpca.4c02482 (PMC11284780; doi:10.1021/acs.jpca.4c02482)
Supplement: Supplementary file 1 — jp4c02482_si_001.pdf [file jp4c02482_si_001.pdf]

Femtosecond to Microsecond Observation of Photochemical Pathways in Nitroaromatic Phototriggers Using Transient Absorption Spectroscopy

William Whitaker,[a] Deborin Ghosh,[a] Partha Malakar,[b] Gabriel Karras,[b] and Andrew J. Orr-Ewing [a,*]

[a] *School of Chemistry, University of Bristol, Cantock's Close, Bristol BS8 1TS, UK*

[b] *Central Laser Facility, Research Complex at Harwell, Science and Technology Facilities Council, Rutherford Appleton Laboratory, Harwell Oxford, Didcot, Oxfordshire, OX11 0QX, UK*

* Author for correspondence: a.orr-ewing@bristol.ac.uk

Contents **Page**

S1 Spectroscopic Methods S3

S2 Steady State Measurements and Density Functional Theory Calculations S5

- **S2.1 Experimental Details for Steady State Measurements** S5
- **S2.2 Comparison of UV-Visible Absorption Spectra with DFT Calculations** S6
- **S2.3 Comparison of FTIR Spectroscopy with DFT Calculations** S8
- **S2.4 TDDFT calculations for NB-Tyr Isomers and Anions** S11

S3 Analysis of Transient Absorption Spectra S12

- **S3.1 Decomposition of TEA Spectra in DMSO** S12
- **S3.2 Decomposition of TVA Spectra in DMSO** S19
- **S3.3 Kinetic Traces and Biexponential Decay Amplitudes for DMNB-Ser in Mixed Solvents** S22

S3.4 Additional Transient Absorption Spectra for NB-Tyr S24

S3.5 Additional Time Constants for NB-Tyr S25

S3.6 Triplet Quantum Yields S27

S1 Spectroscopic Methods

Transient electronic absorption spectroscopy (**TEAS**) and transient vibrational absorption spectroscopy (**TVAS**) experiments were performed using an ultrafast laser system at the University of Bristol, and the LIFEtime facility at the STFC Rutherford Appleton Laboratory. The details of each system are described comprehensively elsewhere,[1-3] with some recent upgrades to the laser system at the University of Bristol highlighted here. The most significant changes to the laser setup are the replacement of the amplified laser system with a Coherent Astrella-USP system, replacement of the spectrometer used for TEAS measurements,[4] and an increase in the motorised delay stage length to extend the maximum possible pump-probe time delays to 4 ns.

For TEAS experiments at the University of Bristol, an amplified 800-nm output beam generated by a pulsed titanium-sapphire laser (Coherent Astrella-USP, 7 W, 1 kHz, 35 fs) was split to generate pump and probe pulses. Wavelength-tuneable UV pump pulses (360 nm for DMNB-Ser) were generated using a Coherent OperA Solo optical parametric amplifier (**OPA**) to give pulse energies of 500 nJ at the sample. In previous measurements for our experimental set-up, the diameter of the pump beam was determined to be 250 μm at the sample using a Thorlabs BP209-Vis/M beam profiler. A synchronised 500 Hz chopper was used to acquire pump-on and pump-off data in sequential shots by blocking alternate excitation pulses, and the time delay between pump and probe pulses was controlled using an optical delay stage in the 360 nm beam line. Approximately 2 % of the 800-nm output from the titanium-sapphire laser was redirected to generate white-light continuum (**WLC**) probe pulses by focussing the beam onto a rastered 3-mm thick calcium fluoride (**CaF_2**) window. WLC probe wavelengths generated in this way spanned the range 350 – 700 nm. WLC probe pulses were recollimated using an off-axis parabolic mirror before being focussed into the sample. Experiments using this apparatus allowed observation of time delays from approximately 100 fs to 3.5 ns.

For transient absorption spectroscopy measurements using the LIFEtime facility, 285-nm and 360-nm UV pump pulses were generated with average powers at the sample of 130 nJ and

400 nJ respectively. Wavelength-tuneable UV pump pulses were generated using a Light Conversion Orpheus HP OPA. For TEAS experiments, two WLC probe pulses were available that covered the ranges 370 – 480 nm and 470 – 920 nm. Visible to near IR (**VIS-NIR**) supercontinuum probe pulses were generated using a Yb-based laser amplifier by focusing a fraction of the fundamental output (1030 nm) into a 4-mm sapphire window (470 – 920 nm probe region), whereas UV-visible probe pulses were instead generated by focusing a fraction of the second harmonic of the laser output (515 nm) into a 4-mm thick CaF_2 window (370 – 480 nm probe region). For TVAS measurements a pair of IR probe pulses each covering approximately 200 cm^{-1} were spatially overlapped at the sample but offset in central wavenumber to give a contiguous probe region of approximately 1400 – 1800 cm^{-1}. IR probe pulses were generated using a Light Conversion Orpheus ONE OPA. To span pump-probe time delays from < 1 ps to tens of microseconds, the LIFEtime system combined an optical delay stage with pulse picking from the dual-amplifier (Light Conversion, Pharos, 15 W, 100 kHz, 260 fs output and Pharos SP, 6 W, 100 kHz, 180 fs). UV pump beam diameters were 100 – 150 μm for TEAS measurements, and 150 – 200 μm for TVAS measurements.

Solutions of nitroaromatic phototriggers (**PT**s) were prepared in dimethyl sulfoxide (**DMSO**, analytical reagent, $\geq$ 99.5 %) and deuterated dimethyl sulfoxide (**DMSO-d6,** 99.9 atom % D) for TEAS and TVAS measurements respectively. For solutions with mixed DMSO and H_2O solvents, H_2O or D_2O (99.9 atom % D) were added stepwise in 1 mL increments to 10 mL solutions of PTs in DMSO or DMSO-d6. Typical concentrations for solutions of DMNB-Ser used in transient absorption spectroscopy experiments were 3 mM, 2.7 mM, and 2.4 mM for solutions containing 10 mL of DMSO and 0 mL, 1 mL and 2 mL of water respectively. Typical concentrations for solutions of NB-Tyr used in transient absorption spectroscopy experiments were 1.6 mM, 1.5 mM, and 1.3 mM for solutions containing 10 mL of DMSO and 0 mL, 1 mL and 2 mL of water respectively. Solutions were flowed through a stainless-steel Harrick cell (250 μm path length) fitted with two CaF_2 windows using a peristaltic pump.

S2 Steady State Measurements and Density Functional Theory Calculations

S2.1 Experimental Details for Steady State Measurements

For steady state spectroscopy measurements, samples of DMNB-Ser (13 mM) and NB-Tyr (2 mM) were prepared in DMSO-d6 and transferred to a stainless-steel Harrick cell for data acquisition. Harrick cells were fitted with two CaF_2 windows that were optically transparent in the spectral regions used, and a PTFE spacer located between the windows defined the sample path length.

Steady state UV-Visible absorption spectra were recorded using a Thermo Scientific Genesys 10S UV-Visible spectrometer at the University of Bristol. Spectra were acquired over the range 200 – 800 nm. Background spectra of DMSO-d6 were acquired using the same Harrick cell as for sample measurements.

FTIR spectra were measured using a PerkinElmer Spectrum Two FTIR spectrometer at the University of Bristol. Spectra were acquired over a typical range of 1000 – 4000 cm^{-1} using a Harrick cell with a 50 μm spacer. Background spectra were measured for an empty Harrick cell, and spectra of DMSO-d6 were measured using the same Harrick cell as for sample measurements. FTIR spectra for samples were solvent-subtracted to account for contributions of the solvent to the spectral profiles.

S2.2 Comparison of UV-Visible Absorption Spectra with DFT Calculations

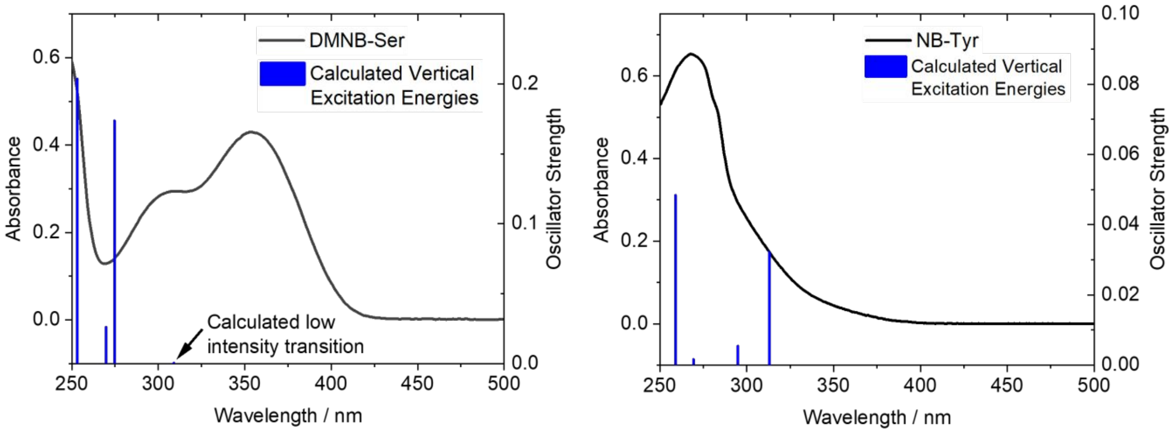

Figure S1. *UV-visible absorption spectra (black lines) of nitroaromatic phototriggers DMNB-Ser (left) and NB-Tyr (right) in DMSO-d6 overlaid with vertical excitation energies (blue bars) calculated at the ωB97XD/6-31+G(d) level of theory using the Tamm-Dancoff approximation. Calculated vertical excitation energies are presented without any scaling.*

The spectra shown in Figure S1 for DMNB-Ser were recorded for a 13 mM solution in a Harrick cell fitted with CaF_2 windows separated by 50 μm. For NB-Tyr the concentration was 2 mM, with a 200 μm window spacing. To ensure that these measurements were not significantly affected by aggregation of the phototrigger solutes, the DMNB-Ser spectrum was also recorded at a concentration of 0.04 mM in a 1-cm quartz cuvette, which revealed the same absorption bands at wavelengths around 300 and 360 nm.

The UV-visible absorption spectrum of DMNB-Ser in DMSO-d6 informed the decision to excite the sample at 360 nm, on the red-edge of the first major absorption band. TDDFT calculations report that this excitation is to the second excited singlet state, which is of $\pi\pi^*$ character, whereas the first singlet excited state, arising at an excitation wavelength of 309 nm, is of $n\pi^*$ character and has negligible oscillator strength. Natural transition orbital (**NTO**) diagrams for DMNB-Ser calculated at the ωB97XD/6-31+G(d) level of theory informed assignment of

excited state electronic character. Assignments of excited state electronic character are consistent with assignments made for nitrobenzene (**NB**) using a high level of theory.[5]

NB-Tyr was excited using 285-nm UV light in order to populate a low-lying singlet excited state of sufficient oscillator strength that strong transient signal is observed. The precise electronic transition induced by a 285-nm laser pulse is difficult to determine from the UV-visible spectrum and DFT calculations, however comparison to experimental and theoretical studies of NB indicates that this is likely to be to either the S_3 or S_4 electronic state.[5, 6] NTO diagrams for the first singlet excited state for NB-Tyr are plotted in figure S2a and show that the S_1 state is of $n\pi^*$ character, which is consistent with the electronic character for the S_1 state of DMNB-Ser, shown in figure S2b. The calculated S_0 to S_1 transition wavelengths for DMNB-Ser and NB-Tyr are similar, appearing at 309 nm and 313 nm respectively.

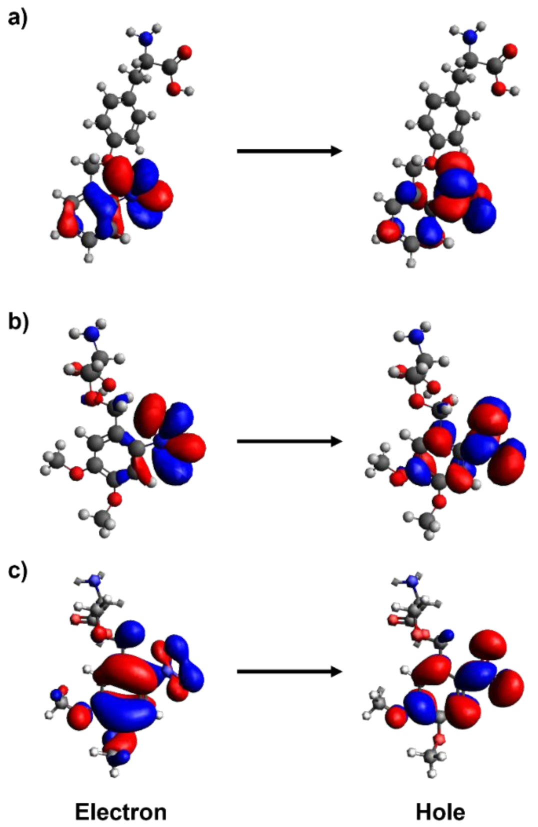

Figure S2. *NTO analysis for the (a) nitro-S_0 to nitro-S_1 $n\pi^*$ transition in NB-Tyr, (b) the nitro-S_0 to nitro-S_1 $n\pi^*$ transition in DMNB-Ser, and (c) the nitro-S_0 to nitro-S_2 $\pi\pi^*$ transition in DMNB-Ser.*

S2.3 Comparison of FTIR Spectroscopy with DFT Calculations

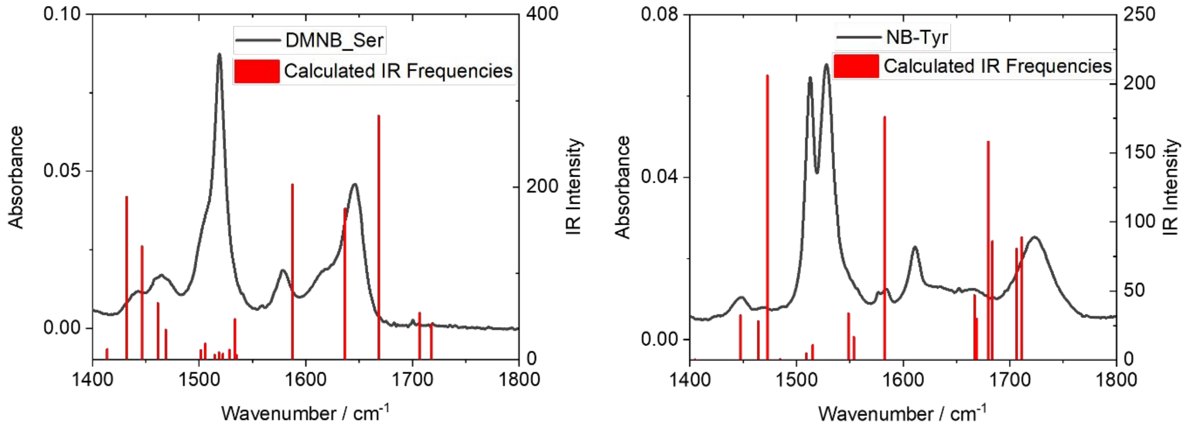

Figure S3. *FTIR spectra (black lines) of nitroaromatic phototriggers DMNB-Ser (left) and NB-Tyr (right) in DMSO-d6, overlaid with vibrational frequencies (red bars) calculated at* the *ω* B97XD/6-31+G(d) level of theory using a DMSO PCM for the *nitro*- isomers of DMNB-Ser and NB-Tyr in their ground electronic states. Calculated IR frequencies are shown without any scaling.

FTIR spectra for nitroaromatic phototriggers overlaid with calculated vibrational frequencies for their ground state *nitro*- isomers are presented in figure S3. Vibrational absorption bands in the FTIR spectra appear at corresponding wavenumbers to negative features in TVAS spectra, confirming that the negative bands in the transient measurements arise because of ground state vibrational bands (ground state bleach features), and not stimulated emission.

Shown in figure S4 are comparisons of vibrational frequencies calculated for ground state isomers of DMNB-Ser and NB-Tyr at the *ω*B97XD/6-31+G(d) level of theory using a polarizable continuum model (**PCM**) to account implicitly for DMSO solvation. Comparison of calculations for DMNB-Ser indicates a cluster of low-intensity vibrational modes between 1490 and 1530 cm^{-1} that are common for all isomers. These modes are assigned to a variety of alkyl bends and stretches, primarily of the methoxy- substituents and CH_2 backbone. There are two unique modes calculated for *nitro*-DMNB-Ser (black bars) at 1570 cm^{-1} and 1605 cm^{-1} that are

assigned to asymmetric NO_2 stretching modes. The NO_2 moiety is unique to the *nitro*-tautomer and is therefore not observed on formation of *aci*- isomers.

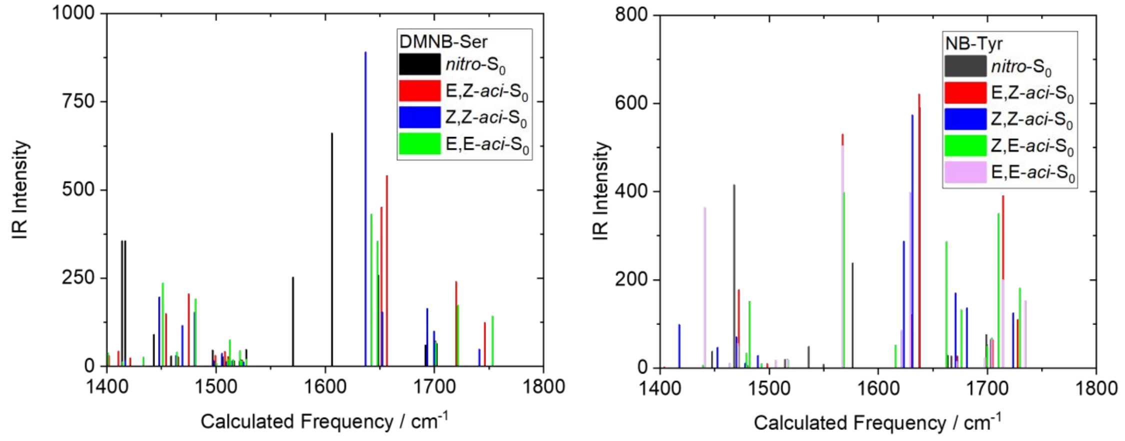

Figure S4 *Calculated vibrational frequencies for ground state optimised structures of nitroaromatic phototriggers and their isomers that are accessible on UV irradiation. Calculated IR frequencies are presented without scaling.*

Despite a discrepancy between the calculated frequencies for NO_2 asymmetric stretching vibrations (1570 – 1605 cm^{-1}) and the experimental band positions of these ground-state vibrational modes in DMNB-Ser (1500 - 1520 cm^{-1}), it is reasonable to assign the bands to these stretching modes. NO_2 modes in organic species are known to manifest as strong absorption bands around 1500 – 1550 cm^{-1} in IR spectroscopy, which is consistent with the position of the peaks we observe for DMNB-Ser. FITR spectra for similar nitroaromatic molecules, 4-nitrophenol and 4-nitocatechol, also display strong absorption bands around 1520 cm^{-1} that are assigned to NO_2 vibrational modes.

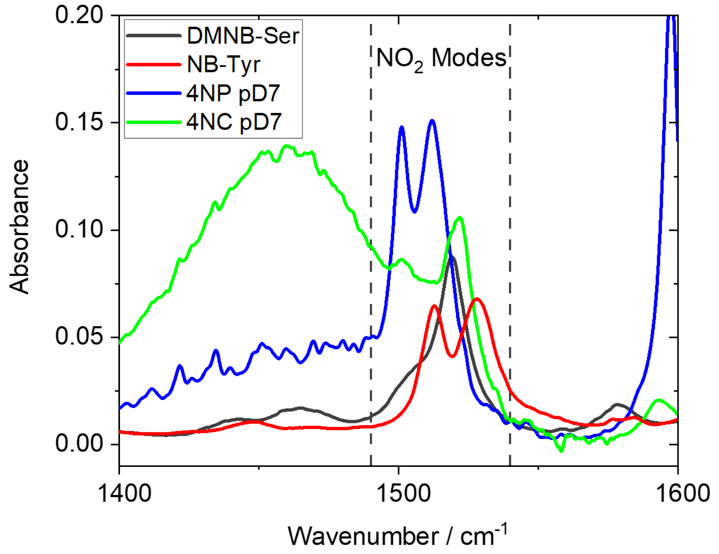

Figure S5. *FTIR spectra (solid lines) for three different nitroaromatic compounds, DMNB-Ser in DMSO-d6 (black), NB-Tyr in DMSO-d6 (red), 4-nitrophenol (4NP) in D_2O at pD 7 (blue), and 4-nitrocatechol (4NC) in D_2O at pD 7 (green). Vertical dashed lines indicate the spectral region across which NO_2 vibrational modes are observed.*

Calculations for NB-Tyr indicate that NO_2 stretching modes occur at frequencies around 1640 - 1670 cm^{-1}, and that these modes are active only in *nitro*- tautomers. As for DMNB-Ser, we assign the 1500 – 1550 cm^{-1} FTIR band to an NO_2 stretching mode, despite a large discrepancy between the experimental FTIR vibrational bands (1500 – 1550 cm^{-1}) and the calculated vibrational frequencies of the NO_2 mode (1700 cm^{-1}).

S2.4 TDDFT Calculations for NB-Tyr Isomers and Anions

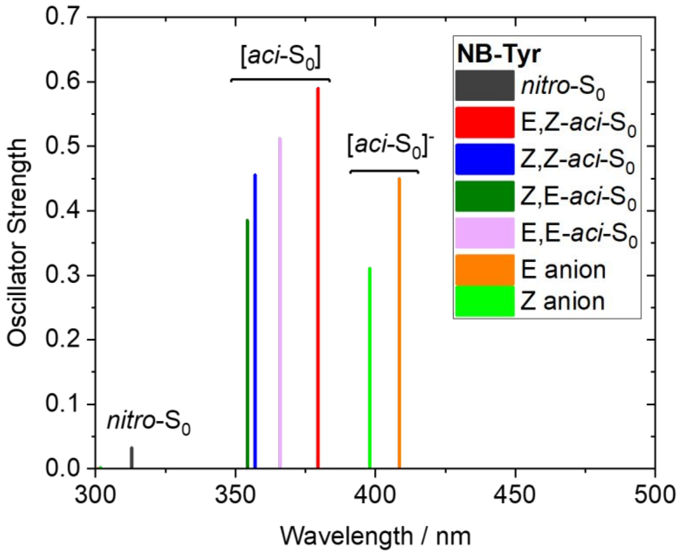

Figure S6. *Computed wavelengths (bars) for the first allowed absorption bands from the ground-state isomers and anions of NB-Tyr, calculated using TDDFT at the ωB97XD/6-31+G(d) level of theory using the Tamm-Dancoff approximation. The inset key provides the colour codes used for the different species. Solvation in DMSO has been described implicitly using the integral equation formalisation of the polarizable continuum model (IEFPCM). Calculations are presented without scaling.*

S3 Analysis of Transient Absorption Spectra

Processing and analysis of transient absorption spectroscopy data were implemented in the KOALA2 programme.[7] For all measurements, except for TEAS measurements of NB-Tyr, a spectrum recorded at negative time delay was subtracted from all spectra obtained at subsequent time delays. Due to the persistence of weak (< 1 mOD) negative ΔA signals between excitation pulses for TEAS measurements of NB-Tyr, negative time subtraction was not performed. A flat-shift baseline correction was applied to all TVAS measurements, but not to TEAS measurements because there are no featureless spectral regions in the data where a baseline level can be established. Corrections to TEAS measurements for DMNB-Ser experiments performed at the University of Bristol were implemented in KOALA2 during analysis to account for frequency chirp in the WLC probe pulse (only evident for time delays up to 500 fs). Chirp correction was not necessary for TVAS measurements, or for TEAS measurements conduced at the LIFEtime facility. Fits to carefully chosen Gaussian functions and basis spectra were used to decompose measured spectra into individual components as described below. Basis spectra were specifically selected to represent individual transient absorption features so that time constants determined from the interconversion of basis spectra reflect the timescales of the corresponding dynamical processes. Time constants were extracted from decomposed spectra by fitting exponential functions to the time-dependent integrated intensities of the Gaussian functions and basis spectra using Origin software.

S3.1 Decomposition of TEA Spectra in DMSO

TEAS measurements for DMNB-Ser in pure DMSO solvent at the University of Bristol using a CaF_2 WLC probe were decomposed using two basis spectra obtained at different delay times. A 0.5 ps basis spectrum was selected to represent the ESA from the *nitro*-S_1 electronic state, and a 1500 ps basis spectrum modelled the ESA from the T_1 electronic state populated by rapid ISC (section 3.2 of the main text). The τ_{S1} time constant was obtained by fitting the time-dependent integrated band intensities of the interconverting basis spectra to a

monoexponential growth or decay function with a shared time component. Figure S7 shows a series of analysis frames at representative time points to illustrate how the basis spectra interacted to yield an overall fit to the measured spectrum. Measurements in this region for mixed DMSO/water solvents used basis spectra in the same way as described here, however time-dependent integrated band intensities were fitted instead to biexponential growth or decay functions.

For determination of sub-ps dynamics, a different decomposition method was used. Initially centred at 420 nm, a Gaussian function that was allowed to shift in central wavelength was used to model the evolution of the ESA arising from the S_2 electronic state, which appears as a high intensity positive signal around 400 nm at early times. The time-dependent integrated band intensity for this component was independently fitted to an exponential decay function, with the initial component of decay describing the depopulation of the S_2 state (τ_{IC} in main text).

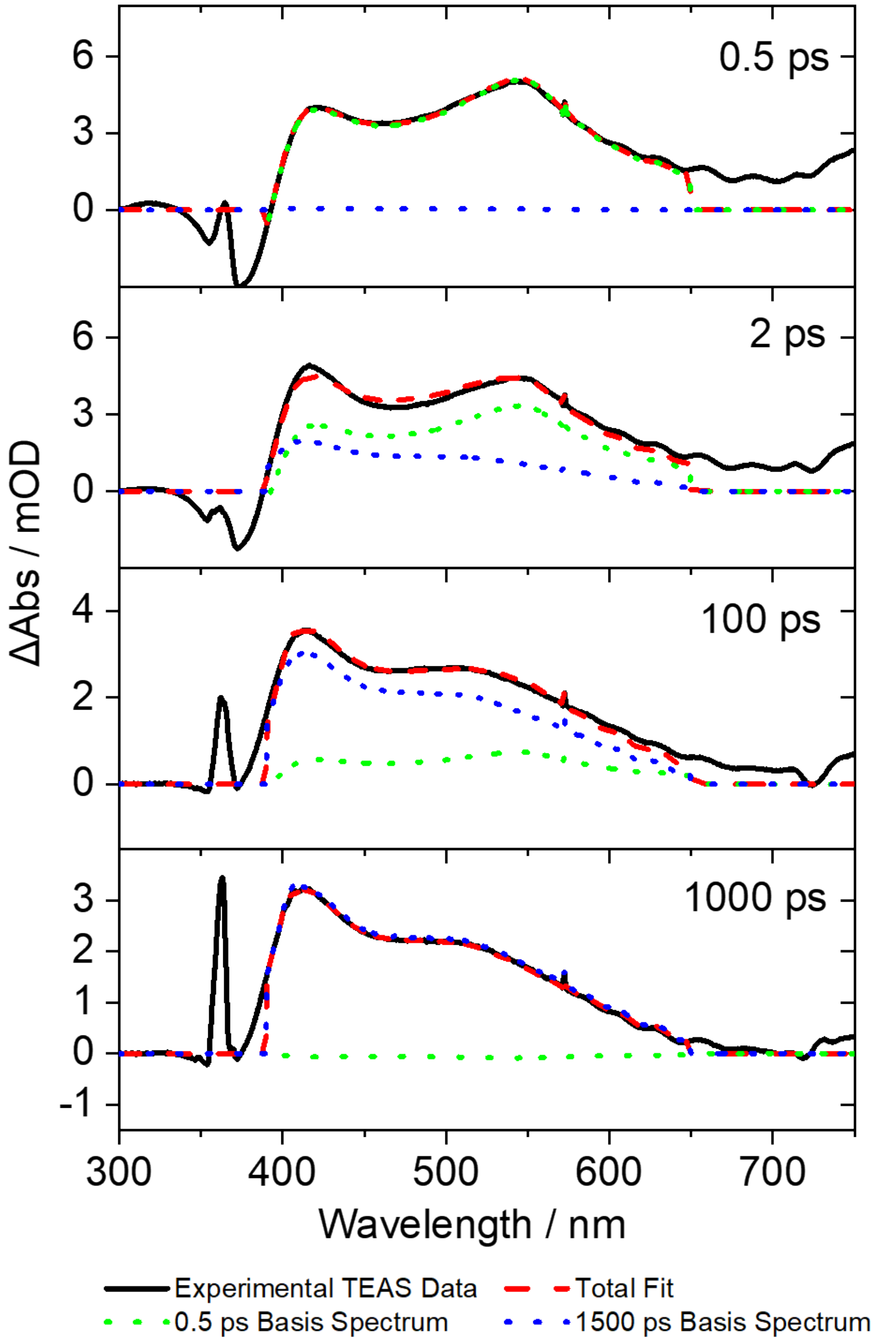

Figure S7. *Example decomposition of TEAS data at several time delays from 0.5 ps to 1000 ps for a solution of DMNB-Ser in DMSO excited at 360 nm. Panels show the experimental TEAS data (black, solid line), the total fit (red, dashed line), and basis spectra used to represent individual electronic states (green and blue dotted lines – as assigned in the key). Delay times for each frame of decomposition are shown in the relevant panels. Spectra are decomposed between 390 nm and 650 nm.*

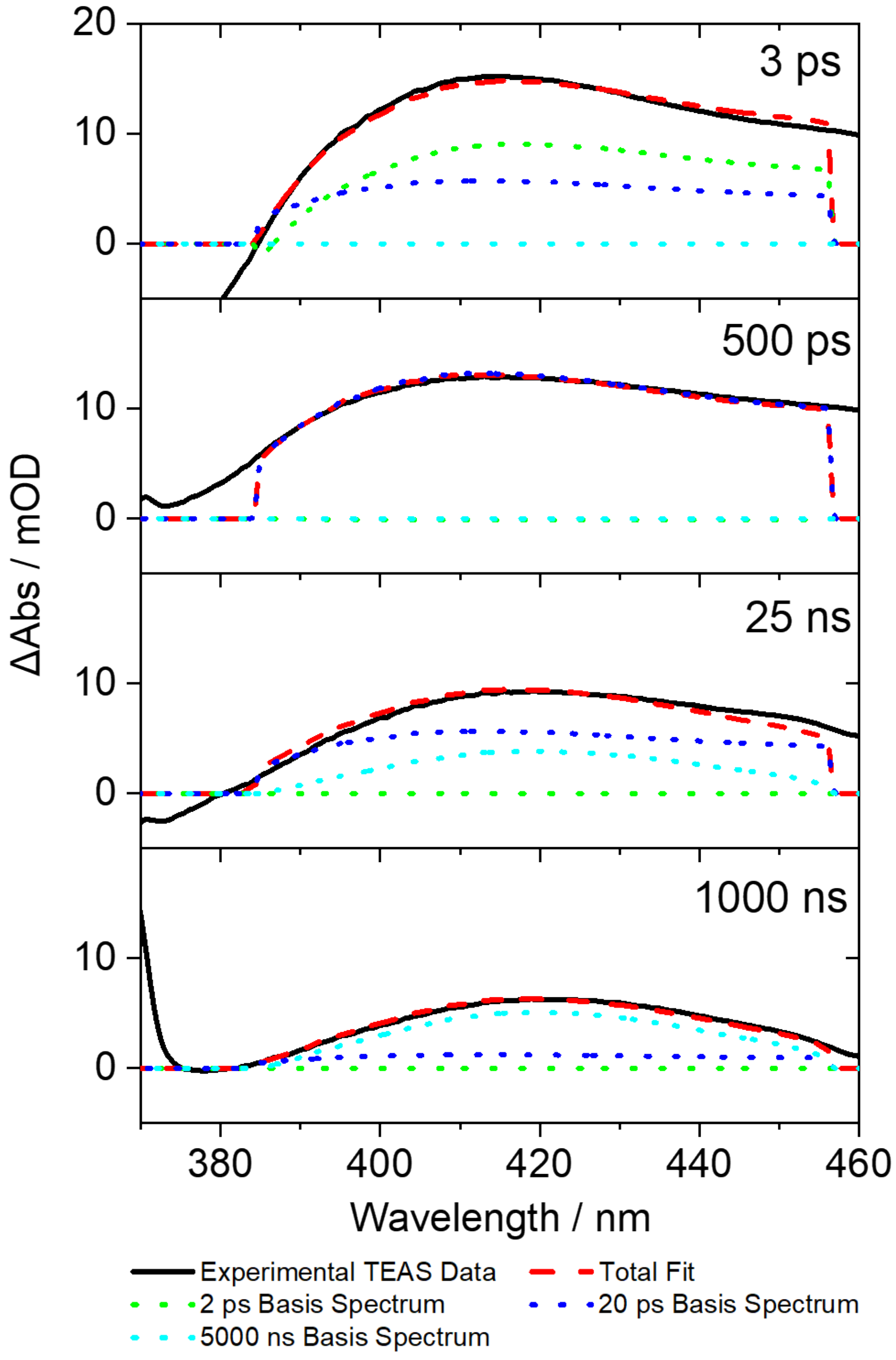

Figure S8. *Example decomposition of TEAS data at several time delays from 3 ps to 1000 ns for a solution of DMNB-Ser in DMSO excited at 360 nm. Panels show the experimental TEAS data (black, solid line), the total fit (red, dashed line), and basis spectra used to represent individual features of the overall spectra (green, blue, and cyan dotted lines – as assigned in the key). Delay times for each frame of decomposition are shown in the relevant panels. Spectra are decomposed between 385 nm and 455 nm.*

Shown in figure S8 are a series of analysis frames representative of the decomposition of TEAS measurements for DMNB-Ser using a 370 – 480 nm probe region at the LIFEtime facility. Due to overlap of spectral features in this region, analysis is divided into two parts that model the spectral evolution over 0 to 500 ps (early time), and 500 ps to 8 μs (late time). For analysis at early time, evolution of the spectra is represented by the interconversion of a 2 ps basis spectrum into a 20 ps basis spectrum. These basis spectra were selected because at 2 ps delay times the *nitro*-S_1 (nπ^*) state is the most significant contributor to the observed ESA, and by 20 ps the spectrum has evolved so that the most significant ESA contributor is a distinct band that we assign to the triplet excited state populated by ISC. A time constant (τ_{S1}) is then extracted by fitting the time-dependent integrated signals of these functions to monoexponential decay or growth functions with a shared time constant. Decomposition of the spectra at late time is similar, but instead analyses the interconversion of a 20 ps basis spectrum (modelling the triplet excited state absorptions) into a 5 μs basis spectrum before fitting the integrated signals to biexponential decay or growth functions with shared time constants. Late time analysis yields τ_D and τ_{Iso} time constants. In this instance the 5 μs basis spectrum reflects the final state of the system observed on our timescales. Therefore, the time constants extracted from the interconversion from the 20 ps to 5 μs basis spectra are informative of the timescales for processes we interpret as deprotonation and isomerization as the population relaxes from the *nitro*- triplet states and most returns to *nitro*-S_0.

Decomposition of TEA spectra for NB-Tyr in the 370 – 480 nm probe region was performed similarly to that for DMNB-Ser, however spectral congestion necessitated dividing the analysis into three parts. Initial decomposition used two basis spectra at selected time delays of 1 ps and 90 ps and modelled the spectral evolution between 0 ns and 1.5 ns delay times. Extracted time-dependent integrated signals of the basis spectra were fitted using biexponential functions, with the first time component shared to yield a value of τ_{S1}, and the second time constant allowed to converge independently to yield τ_{RISC}. Significantly, the amplitude of the second exponential term for the 1-ps basis spectrum was fixed to zero, therefore the τ_{RISC}

time constant was determined using the fits to the 90-ps basis spectrum only. Between 1.5 ns and 1 μs, a 100-ns basis spectrum was used to extract the kinetics of the deprotonation process. The integrated signal for this function was fitted to a monoexponential decay function. Finally, between 1.25 μs and 8 μs, basis spectra corresponding to 1-μs and 8-μs time delays were used to model the observed shift in the ESA band maximum. Fitting the integrated signals of the basis spectra to monoexponential functions with a shared time constant yielded values for the τ_{Iso} time constant.

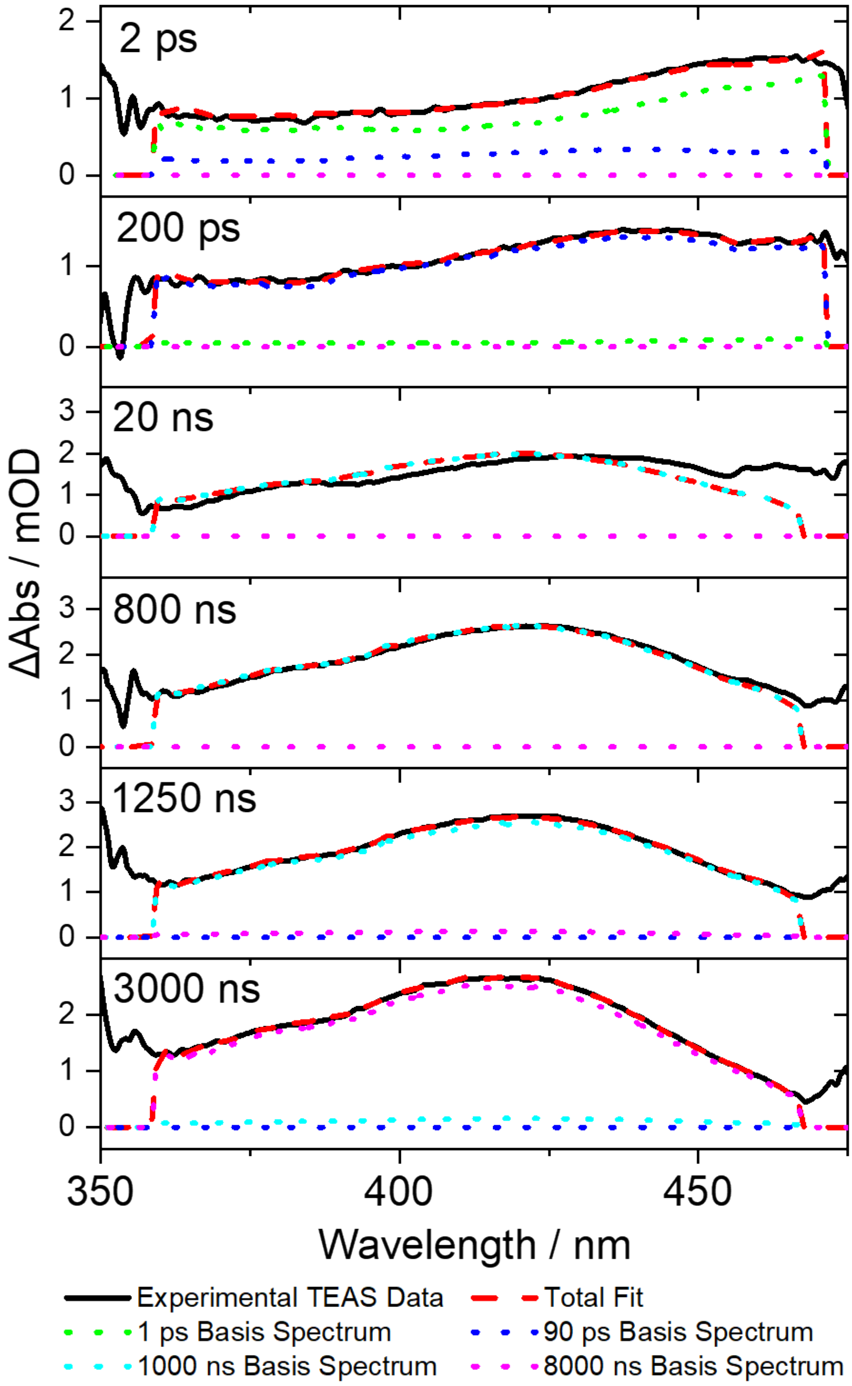

Figure S9 *Example decomposition of TEAS data at several time delays from 2 ps to 3000 ns for a solution of NB-Tyr in DMSO excited at 285 nm. Panels show the experimental TEAS data (black, solid line), the total fit (red, dashed line), and basis spectra used to represent individual features of the overall spectra (green, blue, cyan, and magenta dotted lines – as assigned in the key). Delay times for each frame of decomposition are shown in the relevant panels. Spectra are decomposed between 360 nm and 470 nm.*

S3.2 Decomposition of TVA Spectra in DMSO

A selection of analysis frames for TVAS measurements of DMNB-Ser in DMSO solvent is shown in figure S10. Decomposition of the measured spectra was achieved using two modified basis spectra taken at 2 ps time delay, and three supplementary Gaussian functions. Modification of the basis spectra involved replacing any negative ΔA with zero in one spectrum, and conversely replacing any positive ΔA values with zero in another. The purpose of these changes was to produce basis functions that are representative of the excited state absorptions only (replacing negative ΔA with zero), and of the ground state only (replacing positive ΔA with zero) so that any dependency upon one another is removed and the kinetics can be extracted in isolation. Two Gaussian functions representing the initial and final positions of a shifting ESA feature not properly described by the ESA basis, and a third accounting for an obscured GSB feature, were also included in the decomposition. TVAS time constants were then extracted using a biexponential fit to the time-dependent integrated areas of the fitted basis spectra, and the initial and final ESA Gaussian functions. The kinetics observed for the obscured GSB function were verified to be consistent with the shared biexponential fit for other components of the decomposition, but were excluded from the shared fit due to a low integrated signal.

For TVAS measurements of NB-Tyr, a basis spectrum was created using experimental FTIR data. Inversion about the x-axis and a small linear shift of the FTIR spectrum yielded an appropriate basis spectrum that described the ground state kinetics well. This fitting function was supplemented with two Gaussian functions centred at 1494 cm^{-1} and 1506 cm^{-1} to describe the excited state absorption bands. Fitting of the time-dependent integrated areas of these fitted basis functions to a biexponential decay function yielded TVAS time constants for NB-Tyr.

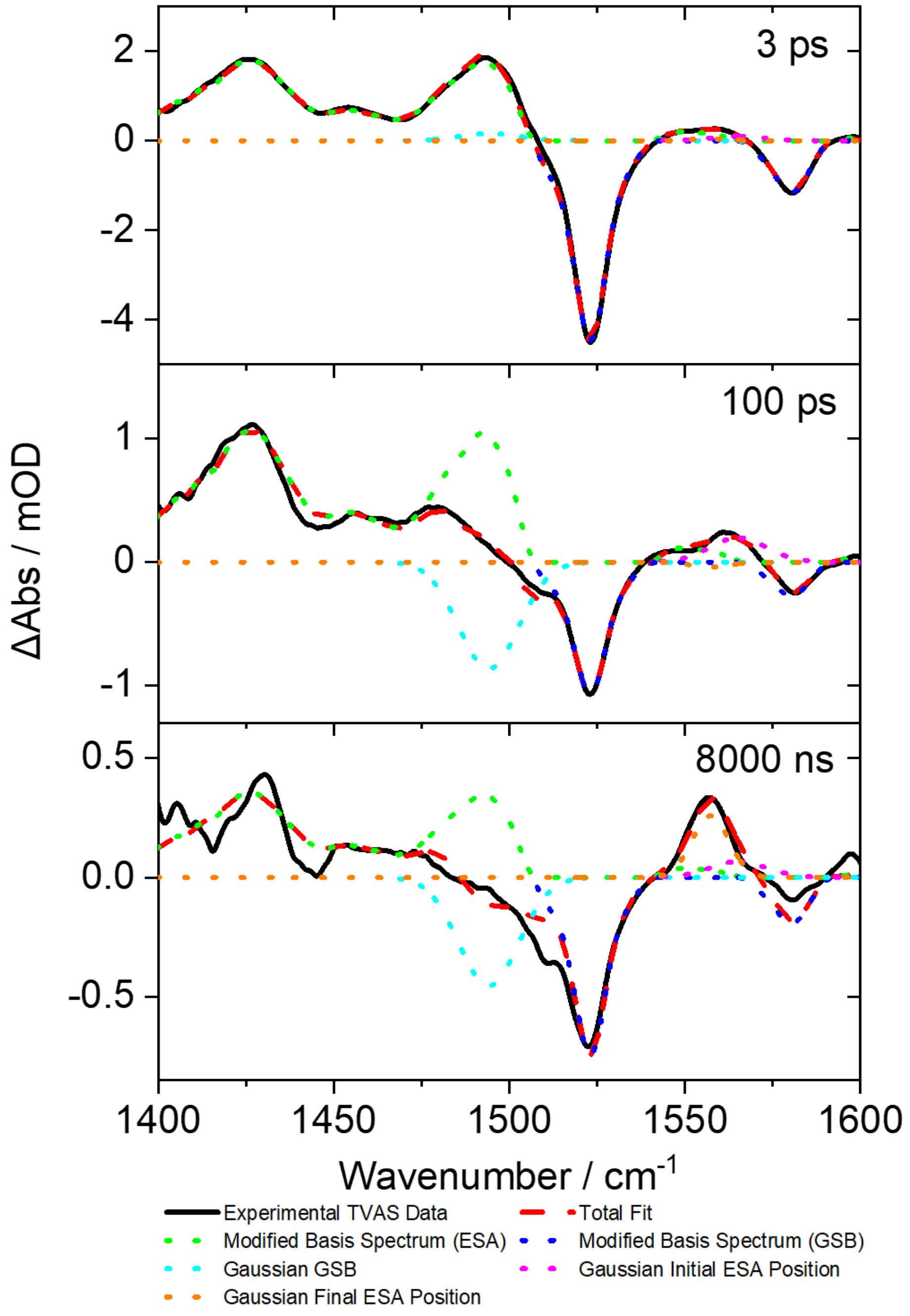

Figure S10. *Example decomposition of TVAS data at several time delays from 3 ps to 8000 ns for a solution of DMNB-Ser in DMSO-d6 excited at 360 nm. Panels show the experimental TVAS data (black, solid line), the total fit (red, dashed line), modified basis spectra used to represent electronic excited states (green dotted line) and the ground state (blue dotted line), and Gaussian functions used to model individual features of the overall spectra (cyan, magenta, and orange dotted lines – as assigned in the key). Delay times for each frame of decomposition are shown in the relevant panels.*

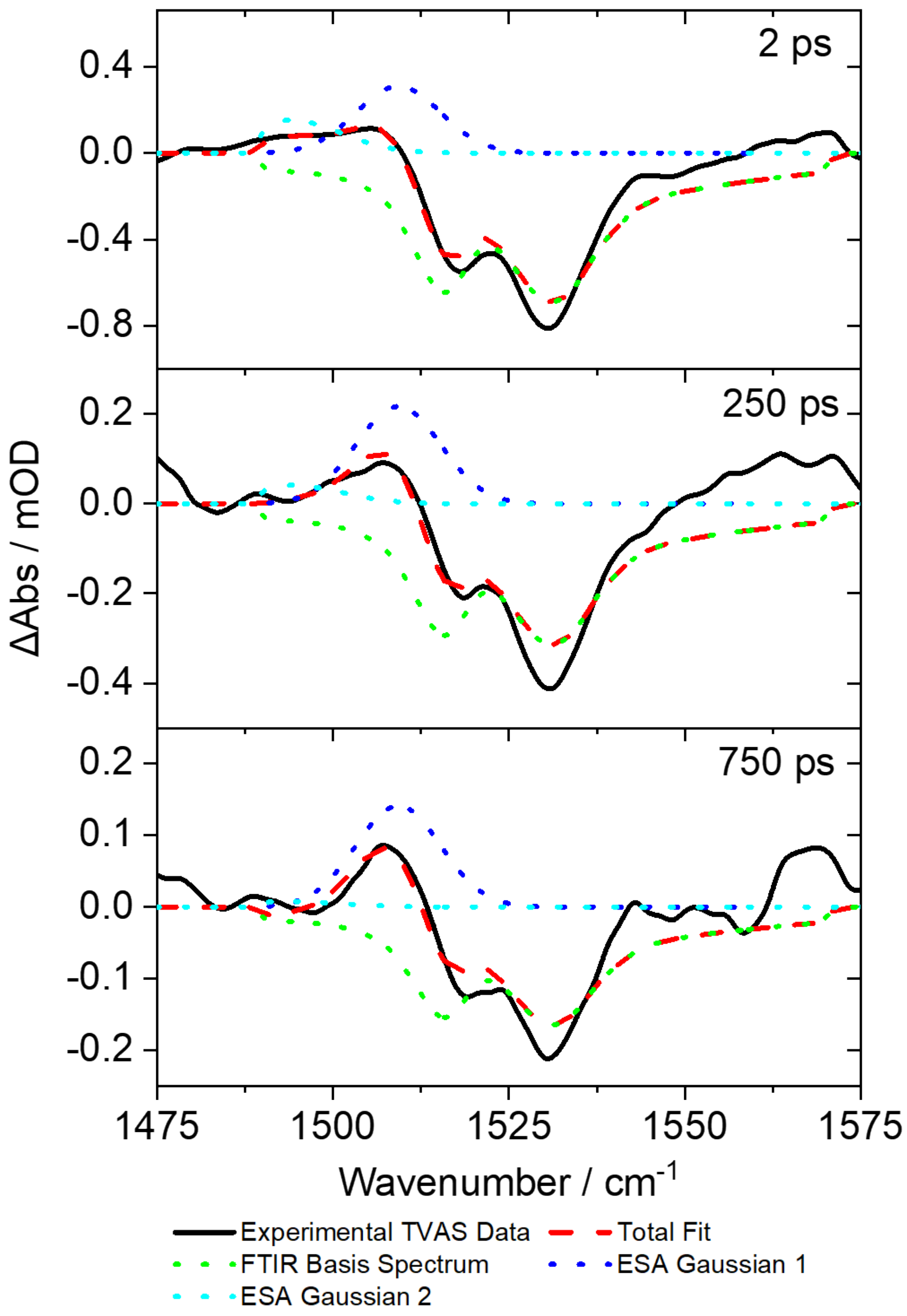

Figure S11. *Example decomposition of TVAS data at several time delays from 2 ps to 750 ps for a solution of NB-Tyr in DMSO-d6 excited at 285 nm. Panels show the experimental TVAS data (black, solid line), the total fit (red, dashed line), an FTIR basis spectrum used to represent the ground electronic state (green dotted line) and Gaussian functions used to model individual features of the overall spectra (blue and cyan dotted lines – as assigned in the key). Delay times for each frame of decomposition are shown in the relevant panels.*

S3.3 Kinetic Traces and Biexponential Decay Amplitudes for DMNB-Ser in Mixed Solvents

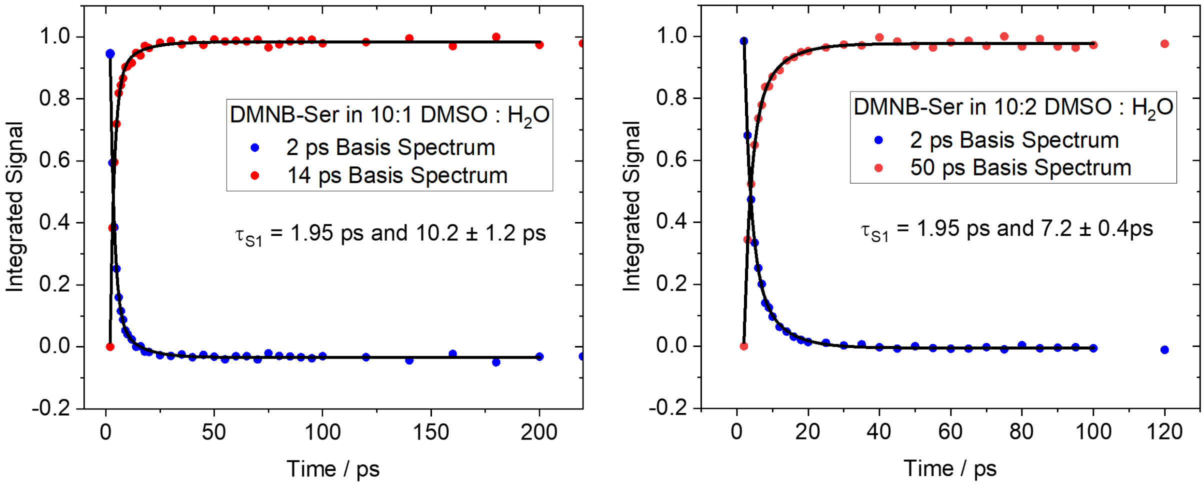

Figure S12. *Kinetic traces for solutions of DMNB-Ser in mixed DMSO/ H_2O solvents for delay times between 0 and 200 ps. Solid black lines are biexponential fits with shared time constants to experimental data (circles). Data points represent the time-dependent integrated signals of basis spectra used in spectral decomposition. Solution composition by volume and determined time constants are shown within the figures.*

Kinetic traces for solutions of DMNB-Ser in mixed DMSO / water solvents are shown in figure S12 for data acquired using a 370 – 480 nm WLC probe pulse. Spectral decomposition for samples in mixed solvents was performed in the same way as described for TEAS decomposition for pure DMSO solvent. For the kinetic traces shown, the interconversion of a 2 ps basis spectrum into 14 ps and 50 ps basis spectra respectively was monitored to describe the simultaneous depopulation of S_1 and population of T_1. Biexponential functions were used to produce fits to the datapoints. For the first time component, a value of 1.95 ps was chosen as it matches the τ_{S1} monoexponential time constant determined for DMNB-Ser in pure DMSO. This time constant was fixed and shared between both fits to the decay of the 2 ps basis function and growth of the second spectral component. The second time constant was also shared in each of the fits to time-dependent datasets, but was unconstrained and allowed to converge to a best-fit value.

Amplitudes of biexponential fits are presented in table S1 for measurements of DMNB-Ser in mixed solvents.

Table S1. Amplitudes of biexponential fitting components for TEAS measurements of DMNB-Ser in mixed solvents.

Probe region	Solvent composition (DMSO : H_2O by volume)	A_1	A_2	A_1 / %
CaF_2	10 : 1	0.37 ± 0.01	0.08 ± 0.01	82 ± 3
	10 : 2	0.56 ± 0.01	0.10 ± 0.01	85 ± 2
515WLC	10 : 1	2.36 ± 0.05	0.17 ± 0.02	93 ± 3
	10 : 2	1.99 ± 0.06	0.38 ± 0.03	84 ± 3
1030WLC	10 : 1	0.54 ± 0.03	0.04 ± 0.03	93 ± 9
	10 : 2	0.38 ± 0.05	0.14 ± 0.05	73 ± 14

For 1 mL of water estimates were made for fitting amplitudes of the S1 basis spectrum. For 2 mL of water estimates were made for fitting amplitudes of the T1 basis spectrum.

S3.4 Additional Transient Absorption Spectra for NB-Tyr

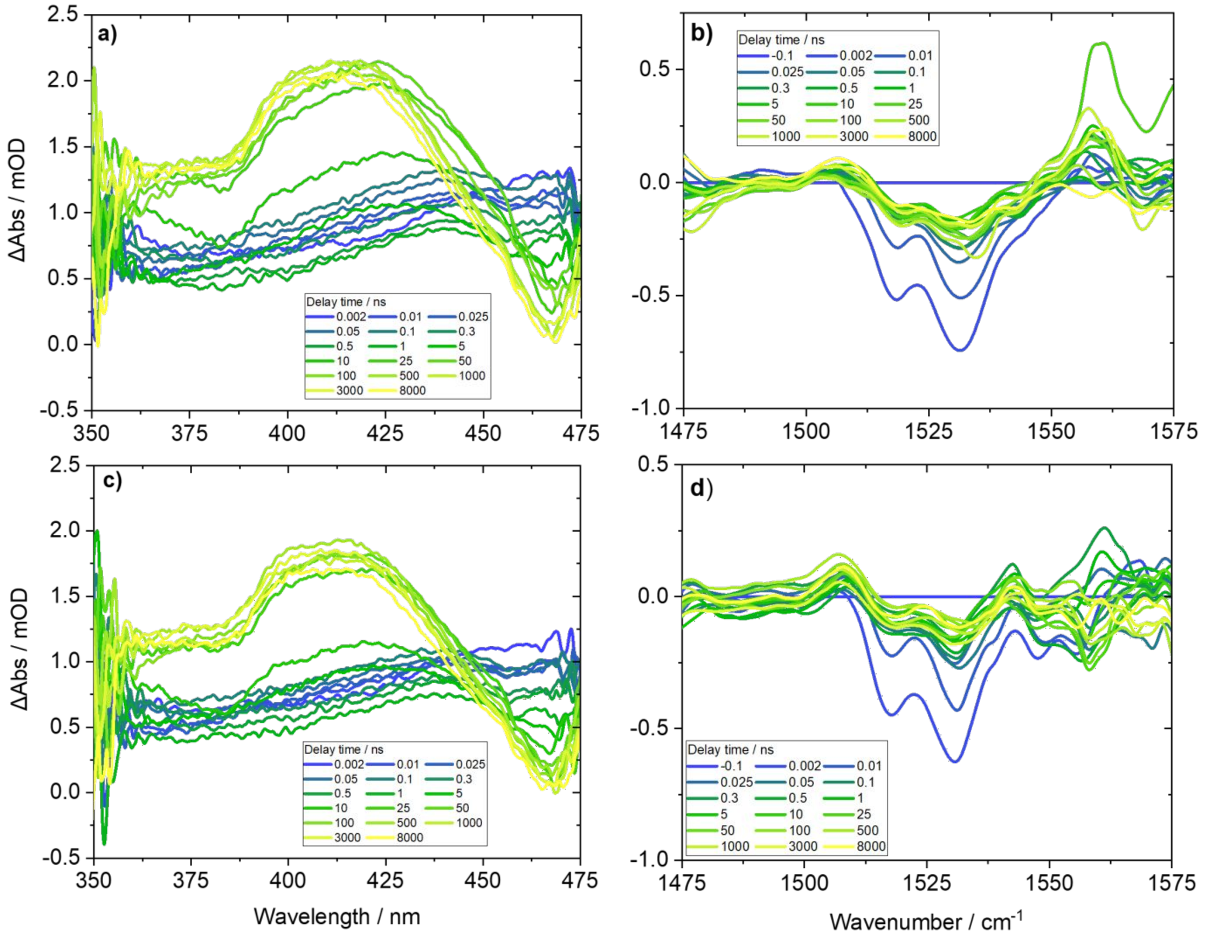

Figure S13. *Transient electronic (a,c) and transient vibrational (b,d) absorption spectra for solutions of NB-Tyr in mixed solvents obtained over 2 ps to 8 μs time delays using a 285 nm UV pump pulse. Solution compositions are (a) 10: 1 DMSO:H_2O, (b) 10: 1 DMSO-d6: D_2O, (c) 10: 2 DMSO: H_2O, and (d) 10: 2 DMSO-d6: D_2O by volume. Spectra are coloured to indicate the delay time of the WLC probe pulse.*

S3.5 Additional Time Constants for NB-Tyr

Table S2. Time constants for NB-Tyr dynamics measured using TEAS and TVAS in mixed solutions of DMSO (10 mL) with added H_2O or D_2O.

Probe Type	V_{water} / mL	Time Constants			
		τ_{S1} / ps	τ_{RISC} / ps	τ_{D} / ns	τ_{Iso} / ns
WLC (370 – 480 nm)	0	13.9 $\pm$ 1.2	346 $\pm$ 57	10.2 $\pm$ 1.5	1010 $\pm$ 160
	1	14	345	10.1 $\pm$ 0.8	2140 $\pm$ 970
	2	14	345	9.3 $\pm$ 0.9	//
IR#	0	11.9 $\pm$ 0.6	246 $\pm$ 32	//	//
	1	10.4 $\pm$ 0.8	265 $\pm$ 70	//	//
	2	9.6 $\pm$ 1.0	702 $\pm$ 250	//	//

# For TVAS measurements, time constants directly report on GSB recovery dynamics.

Time constants for NB-Tyr dynamics following 285 nm UV excitation are summarized in table S2. Time constants were determined by fitting the time-dependent integrated signals of representative basis spectra to exponential functions as described in section S3.1. For τ_{S1} and τ_{RISC} in mixed solvents, analysis showed that the kinetics traces extracted from TEAS measurements were appropriately described by biexponential functions where the τ_1 and τ_2 time components (corresponding to τ_{S1} and τ_{RISC}) are the same as those determined for samples in pure DMSO. For analysis of sub-nanosecond dynamics in TEAS measurements for mixed solvents, biexponential functions were used as described in section S3.1, however the time components $\tau_1$and τ_2 were fixed to 14 ps and 354 ps respectively and are therefore presented without uncertainty.

Table S2 shows that there is no significant change in the τ_{S1} or τ_{RISC} time constants for TEAS or TVAS measurements on addition of water to the solvent, therefore indicating that water does not have an influence on the sub-nanosecond dynamics of NB-Tyr. This observation contrasts with behaviour observed for DMNB-Ser where addition of water yields a second, longer component to the *nitro*-S_1 ($n\pi^*$) lifetime on the order of tens of picoseconds, attributed to statistical interaction of the solute with water in the solvent (section 3.5 of the main text). For DMNB-Ser, the τ_{S1} time constant in pure DMSO is around 2 ps, but on addition of water, a 10 ps time constant is also observed. For NB-Tyr the τ_{S1} time constant in DMSO is 14 ps, therefore it is unlikely that interaction with water in a mixed solvent will have a significant effect on the observed S_1 lifetime as it is already on the order of tens of picoseconds. Simply, the rate of depopulation of the *nitro*-S_1 will not be significantly reduced in mixed solutions compared to pure DMSO, and therefore the influence of water is not directly observed.

S3.6 Triplet Quantum yields

As is discussed in the main text, from the *nitro*-S_1 electronic state the excited phototrigger population can undergo ESIHT and subsequent IC to repopulate the electronic ground state, or competitive ISC to populate the triplet manifold. The quantum yield of triplet formation ($\boldsymbol{\Phi_T}$) can be estimated from TVAS measurements using equation S1.

$$\Phi_T = \frac{\Delta Abs(GSB)_{R1}}{\Delta Abs(GSB)_{max}} \qquad \textbf{(Equation S1)}$$

In this equation, $\Delta Abs(GSB)_{max}$ is the change in absorbance of the NO_2 vibrational mode ground-state bleach feature (section S2.3) at its maximum amplitude, and $\Delta Abs(GSB)_{R1}$ is the change in absorbance for this same GSB feature after the first component of GSB recovery (R1) is complete. It is important to note that equation S1 assumes that IC on the ESIHT potential to the ground state is 100 % efficient. Given that the IC pathway is greatly favoured over formation of *aci*-S_1 species via direct *nitro*-S_1 to *aci*-S_1 tautomerism, this assumption should yield reasonable estimates for Φ_T. Furthermore, because the amplitudes of recovery are compared for a single transient absorption feature (here, GSB recovery) on different timescales, the transition dipole moment is constant. Therefore, any effects of changes in transition dipole moments for different spectroscopic bands are not relevant for estimates of Φ_T.

Using TVAS measurements, it is also possible to quantify the proportion of excited state population which undergoes ISC and that does not reform the ground state during the second step of GSB recovery dynamics (R2). These species will exist as intermediates or photoproducts on our timescales. This proportion is referred to here as the "branching yield" and is given by equation S2.

$$Branching\ yield = \frac{\Delta Abs(GSB)_{R2}}{\Delta Abs(GSB)_{R1}}$$ **(Equation S2)**

Finally, the overall quantum yield of photoproduct formation (Φ_P) can be estimated as the product of Φ_T and the branching yield and corresponds to the fraction of initially excited species that do not reform the ground state parent PT molecule on our timescales.

Table S3. Estimates from TVAS measurements of quantum yield of triplet formation, branching yield, and quantum yield of photoproduct formation for DMNB-Ser for excitation using a 360 nm UV pump pulse.

Volume of D_2O in the solvent / mL	Φ_T	Branching yield	Φ_P
0	0.30	0.44	0.13
1	0.25	0.29	0.07
2	0.24	0.21	0.05

Table S4 Estimates from TVAS measurements of quantum yield of triplet formation, branching yield, and quantum yield of photoproduct formation for NB-Tyr on excitation using a 285 nm UV pump pulse.

Volume of D_2O in the solvent / mL	Φ_T	Branching yield	Φ_P
0	0.35	0.58	0.20
1	0.38	0.57	0.22
2	0.36	0.50	0.18

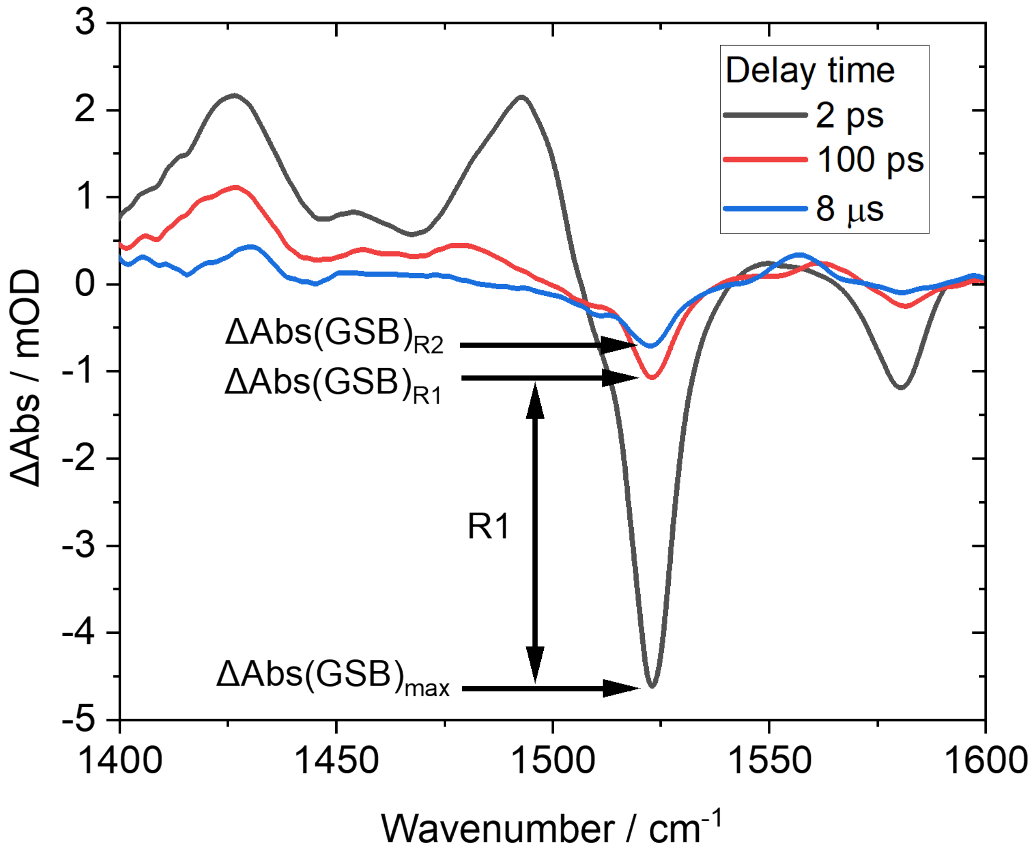

Figure S14 *TVAS data recorded at three time delays (solid black, red and blue lines) for a solution of DMNB-Ser in DMSO-d6. Black, single-headed arrows indicate the positions where the amplitude of the change in absorbance of the NO_2 vibrational mode GSB features were measured for $\Delta Abs(GSB)_{max}$, $\Delta Abs(GSB)_{R1}$ and $\Delta Abs(GSB)_{R2}$, as labelled. The black, double-headed arrow indicates the degree of GSB recovery during the initial component of relaxation (R1). The amplitude of the NO_2 vibrational mode GSB feature after 100 ps (red line) relative to after 2 ps (black line) is representative of the proportion of the excited state population that formed triplet excited states compared to reforming the ground electronic state. The degree of GSB recovery during the second component of relaxation (R2) is shown as the difference between the amplitude of $\Delta Abs(GSB)_{R1}$ and $\Delta Abs(GSB)_{R2}$.*

References:

1. Roberts, G. M.; Marroux, H. J. B.; Grubb, M. P.; Ashfold, M. N. R.; Orr-Ewing, A. J., On the Participation of Photoinduced N-H Bond Fission in Aqueous Adenine at 266 and 220 nm: A Combined Ultrafast Transient Electronic and Vibrational Absorption Spectroscopy Study. *J. Phys. Chem. A* **2014,** *118* (47), 11211-11225.
2. Greetham, G. M.; Sole, D.; Clark, I. P.; Parker, A. W.; Pollard, M. R.; Towrie, M., Time-resolved multiple probe spectroscopy. *Rev. Sci. Instrum.* **2012,** *83* (10), 103107.
3. Greetham, G. M.; Donaldson, P. M.; Nation, C.; Sazanovich, I. V.; Clark, I. P.; Shaw, D. J.; Parker, A. W.; Towrie, M., A 100 kHz Time-Resolved Multiple-Probe Femtosecond to Second Infrared Absorption Spectrometer. *Appl. Spectrosc.* **2016,** *70* (4), 645-653.
4. Kao, M.-H.; Venkatraman, R. K.; Ashfold, M. N. R.; Orr-Ewing, A. J., Effects of ring-strain on the ultrafast photochemistry of cyclic ketones. *Chem. Sci. J.* **2020,** *11* (7), 1991-2000.
5. Giussani, A.; Worth, G. A., Insights into the Complex Photophysics and Photochemistry of the Simplest Nitroaromatic Compound: A CASPT2//CASSCF Study on Nitrobenzene. *J. Chem. Theory Comput.* **2017,** *13* (6), 2777-2788.
6. Lau, N.; Ghosh, D.; Bourne-Worster, S.; Kumar, R.; Whitaker, W.; Heitland, J.; Davies, J. A.; Clark, I. P.; Karras, G.; Greetham, G. M., et al., Unraveling the Ultrafast Photochemical Dynamics of Nitrobenzene in Aqueous Solution. *J. Am. Chem. Soc.* **2024**, 10.1021/jacs.3c13826.
7. Grubb, M. P.; Orr-Ewing, A. J.; Ashfold, M. N. R., KOALA: A program for the processing and decomposition of transient spectra. *Rev. Sci. Instrum.* **2014,** *85* (6), 064104.
